# In situ motions of individual inner-hair-cell stereocilia from stapes stimulation in adult mice

**DOI:** 10.1038/s42003-021-02459-6

**Published:** 2021-08-11

**Authors:** Yanli Wang, Charles R. Steele, Sunil Puria, Anthony J. Ricci

**Affiliations:** 1 Otolaryngology–HNS, Stanford University, Stanford, CA USA; 2 Mechanical Engineering, Stanford University, Stanford, CA USA; 3 Massachusetts Eye and Ear, Harvard Medical School, Boston, MA USA

**Keywords:** Hair cell, Neurophysiology, Cochlea, Transduction

## Abstract

In vertebrate hearing organs, mechanical vibrations are converted to ionic currents through mechanoelectrical-transduction (MET) channels. Concerted stereocilia motion produces an ensemble MET current driving the hair-cell receptor potential. Mammalian cochleae are unique in that the tuning of sensory cells is determined by their mechanical environment and the mode of hair-bundle stimulation that their environment creates. However, little is known about the in situ intra-hair-bundle motions of stereocilia relative to one another, or to their environment. In this study, high-speed imaging allowed the stereocilium and cell-body motions of inner hair cells to be monitored in an ex vivo organ of Corti (OoC) mouse preparation. We have found that the OoC rotates about the base of the inner pillar cell, the hair bundle rotates about its base and lags behind the motion of the apical surface of the cell, and the individual stereocilia move semi-independently within a given hair bundle.

## Abstract (web summary)

Wang et al. use high-speed imaging to examine the in situ movement of inner hair-cell stereocilia in the mouse ear. They report that these individual cilia move semi-independently within their given bundle, which forms part of a larger complex within the inner ear, providing unique filtering opportunity for hearing in mammals.

## Introduction

Hearing brings the world to life, enabling spoken communication and the emotional power of music. In mammals, the transduction of sound into a neural code occurs within the spiral-shaped fluid-filled cochlea^1^. Prior to reaching the cochlea, sound is transduced from an airborne wave into mechanical vibrations of three middle-ear bones. The last bone, the stapes, is attached to the oval window of the cochlea, whose motion establishes a traveling wave within the cochlea^2^.

**Fig. 1:**
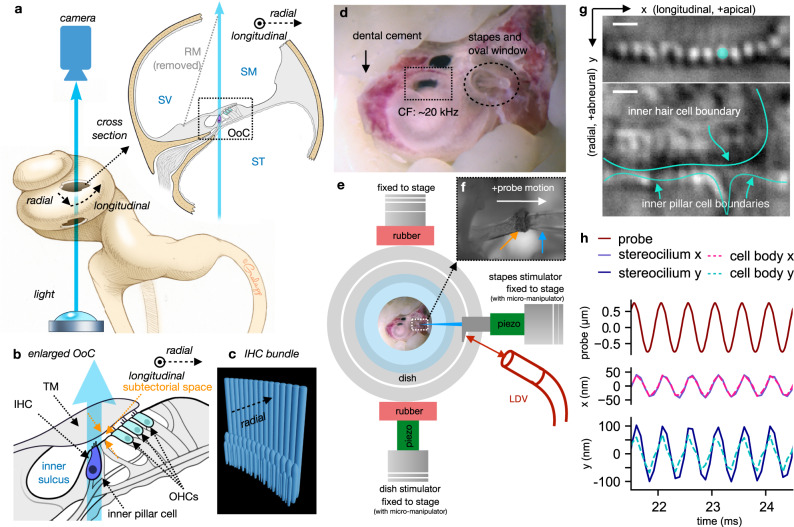
Experimental set-up. **a** A schematic illustration (bottom left) shows the bright-field-microscopy light path (blue arrow) through two holes in the cochlear wall, with an enlarged cross section of the imaging region (upper right) showing the basilar membrane (BM), organ of Corti (OoC), and three fluid chambers: the scala tympani (ST), scala vestibuli (SV), and scala media (SM). The Reissner’s membrane (RM) is removed to improve visibility. **b** An enlarged view of the OoC cross section shows the tectorial membrane (TM), the fluid-filled inner sulcus and subtectorial space, an inner hair cell (IHC) and inner pillar cell, and three outer hair cells (OHCs). **c** A 3-D sketch of an IHC bundle viewed slightly askew from the side. An IHC bundle consists of three stereocilium rows of decreasing height protruding from the apical surface of the cell. **d** The prepared cochlea is shown through a dissection microscope after securing it to the dish with dental cement. The opening in the cochlear wall (the dotted rectangle) is placed around the 20-kHz characteristic frequency (CF) location. **e** A schematic diagram shows the specimen placed on a dish, with piezoelectric stimulation provided through the stapes stimulator via a glass probe or through the dish stimulator. The probe motion is monitored by a laser Doppler vibrometer (LDV). **f** The glass probe (blue arrow) pushes against the stapes head (orange arrow) to provide stimulation, as shown through a ×4 objective lens. The positive direction of the probe motion is defined as moving away from the cochlea. **g** The upper image shows the tallest stereocilium row of an IHC bundle near the tops of the stereocilia, and the lower image outlines in cyan the boundaries of the corresponding IHC cell body and nearby inner pillar cells (9 μm beneath the upper image), all through a ×100 1.0-NA objective lens with ×2 magnifier. Motion of the cyan-colored stereocilium in the upper image is shown in **h**. Both scale bars indicate 1 μm. **h** Motion plots are shown of the stapes probe (upper) and of the example stereocilium and IHC cell body in the longitudinal (*x*; middle) and radial (*y*; lower) directions.

The cochlea is divided longitudinally by the basilar membrane (BM) and Reissner’s membrane (RM) into three fluid chambers (Fig. 1a): the scala tympani (ST), scala media (SM), and scala vestibuli (SV)^1^. Attached to the BM between the SM and ST is the organ of Corti (OoC; Fig. 1b), the epithelium housing one row of sensory inner hair cells (IHCs) and three rows of electromotile outer hair cells (OHCs)^1^. “Hair cells” are named for their sensory organelle, the apically protruding hair bundle (Fig. 1b, c). Hair bundles are comprised of specialized actin-filled microvilli-like stereocilia^3^. Each hair bundle consists of an array of stereocilia organized into 3–4 rows, with 10–20 stereocilia per row (Fig. 1c). The height of the stereocilium rows decreases like a staircase (Fig. 1c). The mechanoelectrical transduction (MET) machinery is housed at the top of the shorter stereocilium rows and is mechanically coupled to the next-tallest stereocilium row via an extracellular tip link. Tip links provide directional sensitivity to the hair bundle, such that deflection of the bundle toward the taller stereocilium rows pulls on the tip links and thus opens the MET channels, whereas bundle deflection in the opposite direction reduces tension in the tip links and thus closes the MET channels^4–6^. The hair bundles are overlaid by the acellular tectorial membrane (TM)^7^, which affects how both the IHC and OHC hair bundles are stimulated^8,9^ (Fig. 1b, c). Sound-induced stapes vibrations cause a traveling wave in the cochlea that yields differential vibrations in the BM, OoC, and TM. The differences in motion of these structures induce stereocilium motions relative to the apical surface of the hair cell, which in turn regulate MET-channel openings. Currents generated by the MET channels produce a hair cell’s receptor potential, which drives synaptic release in IHCs or electromotility in OHCs^10,11^.

Mammalian cochlear hair cells rely on the mode of hair-bundle stimulation, as imparted by the mechanics of the surrounding environment, to establish a receptor potential. IHC hair bundles are generally believed to be freestanding^12,13^ (but see ref. ^14^ for a recent alternative view), so the details of how the IHC stereocilia are stimulated within the subtectorial fluid space remain unclear but are critical to our understanding of information transfer (Fig. 1b). Confounding our understanding further is the finding that IHC hair bundles lack within-bundle cohesiveness, such that individual stereocilia within a bundle move non-uniformly during in vitro experiments^15–17^ in which artificial stimuli involving either glass probes or fluid jets were used, with the TM removed. Because the motion of an individual stereocilium in the tallest row stimulates only a few MET channels in the adjacent shorter rows^18,19^, the manner in which the entire group of tallest-row stereocilia move relative to one another will dictate the timing of ion-channel activation and thereby shape each IHC’s ensemble current and receptor-potential response. Thus, it is critical to determine how individual stereocilia move in response to natural stimulation within the intact OoC, as this will provide direct evidence as to how the receptor potential is shaped, and also provide evidence as to how the IHC hair bundles are stimulated. We used high-speed imaging to investigate: (1) the magnitude and phase of individual stereocilium motion within a bundle, (2) the translational and pivoting motions of IHC hair bundles relative to their apical surfaces, and (3) the cell-body motion to determine how the IHCs move in relation to the base of the pillar cells.

## Results

### Overview of set-up and control experiments

**Fig. 2:**
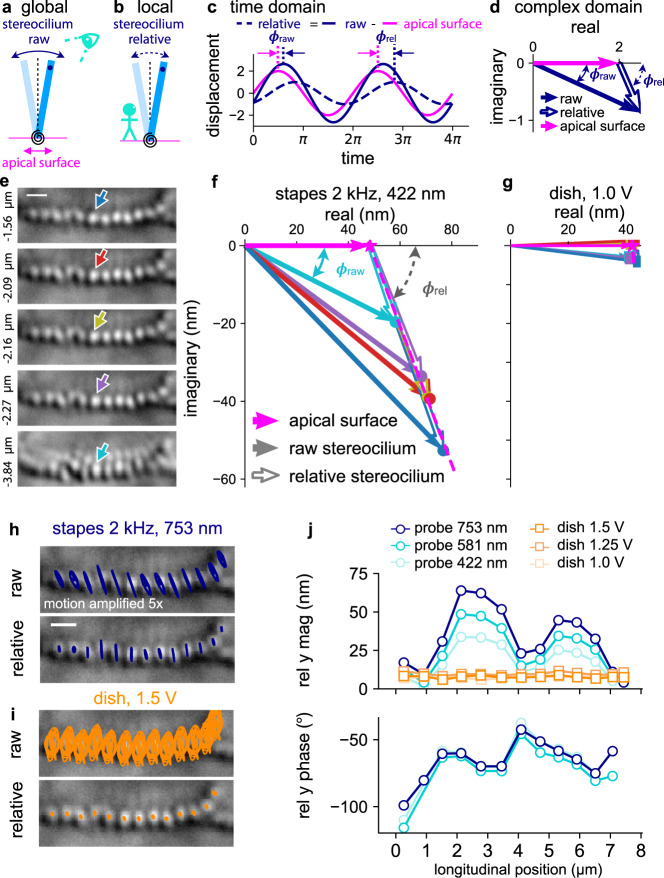
Calculating relative stereocilium motion. **a** The raw motion at the top of a stereocilium is the sum of the stereocilium motion relative to the apical surface and the apical-surface motion. **b** The relative motion of the top of a stereocilium with respect to the apical surface is the difference between the raw motion and the apical-surface motion. **c** A hypothetical time-domain plot shows the raw motions of a stereocilium and the apical surface, with the relative stereocilium motion obtained by subtracting the apical-surface motion from the raw stereocilium motion. **d** The time-domain information from **c** is illustrated in the frequency domain as vectors in the complex plane. The apical-surface motion serves as the zero-phase reference, with the raw stereocilium-motion phase shown as *ϕ*_raw_ and the relative-motion phase as *ϕ*_rel_. **e** A series of images shows an example bundle at different depths indicated to the left of each image. The 0-μm depth position is not precisely controlled but is roughly at the top of the stereocilia where the TM is visible and the bundles are blurry. An example stereocilium is singled out using a differently colored arrow at each depth. The scale bar indicates 1 μm. **f**, **g** Complex-amplitude plots of the motions in the radial direction of the example stereocilium in **e** are shown for a 2-kHz stapes stimulation (**f**) and for dish stimulation (**g**), with the stereocilium-motion vectors color-coded according to the depth in **e**. The points of the raw stereocilium-motion vectors from different depths are fitted with a straight line (dashed magenta; *R*^2^ = 0.97), whose intersection with the real axis indicates the calculated apical-surface-motion vector. The relative-motion vectors are shown as hollow arrows. **h**, **i** For the example bundle of **e**–**g**, the trajectories of the raw (upper) and relative (lower) stereocilium motion (scaled up 5×) are superimposed on top of the original image for stapes stimulation (**h**) and 2-kHz dish stimulation (**i**), with a 1-µm scale bar. **j** The radial-direction relative-motion magnitudes (upper) and phases (lower) relative to the apical surface for each stereocilium are plotted against the longitudinal position of the stereocilium. The relative-motion phases for dish stimulation are omitted from the plot because the phase calculations are error-prone for close-to-zero motions.

We developed a new way of preparing mouse specimens (FVB strain, postnatal age of 20–21 days of either sex) to image OoC motion in situ, with sufficient resolution to visualize a single IHC stereocilium. About halfway along the length of the cochlea, near the 20-kHz characteristic frequency (CF)^20^, a light path through the OoC was created by opening two holes through the bony cochlear wall (Fig. 1a): one on the ST side, to allow for illumination, and the other on the SV side, to allow for bright-field imaging of the stereocilia (Fig. 1g, upper panel). Motion was captured at 12,500 frames per second (fps) as the stapes was stimulated via a glass probe attached to a piezoelectric actuator (Fig. 1e, f). The recordings were repeated at 3 or 4 depths along the length of the stereocilia and at a depth of 3–5 microns below the apical surface of the IHC where the cell boundary becomes more clearly visible (Fig. 1g lower panel). From these images, the “raw” stereocilium motion at multiple depths and the cell-body motion, directly captured by the camera, were extracted at the stimulation frequency of either 2 or 3 kHz. Example displacements of the raw motion are shown in Fig. 1h for results in the longitudinal (middle) and radial (bottom) directions for one stereocilium (solid lines) and the cell body (dashed lines). With these raw measurements, we calculated the motion of the IHC apical surface where the stereocilia pivot (Fig. 2e–g). The “relative” stereocilium motion was then calculated by subtracting the apical-surface motion from the raw stereocilium motion (Fig. 2d, f).

One potential concern of the motion-detection algorithm is that its sensitivity may be affected by variations in the shape and brightness of different stereocilia within the same bundle. To explore this question, we performed a control experiment to induce a close-to-rigid-body motion of the whole cochlea at once by moving the entire dish (Fig. 1e). For each specimen and each field of view, both the stapes (experimental) and dish (control) stimulations were performed, and the same motion-detection algorithm was used on both data sets. A comparison of the magnitude and phase of the raw inter-stereocilium motion differences between the dish- and stapes-stimulation experiments (paired *t* test *p* < 0.001 for all cases) indicates that the inter-stereocilium motion differences observed within a bundle during stapes stimulation were not due to differences of appearance of the individual stereocilia (Fig. S1).

Another potential concern is that the inter-stereocilium differences in magnitude in the radial and longitudinal directions could be affected by different inclination angles of the stereocilia relative to the imaging plane, combined with stereocilium motion along the optical axis during stimulation (Fig. S2). We found no correlation between the inclination angle and motion magnitude of the stereocilia, indicating that the effect of the stereocilium inclination angle is not relevant (Fig. S2).

### Motion of individual stereocilia relative to the hair-cell apical surface

We assume that the pivoting points of the stereocilia (rotational springs in Fig. 2a, b) are close to the apical surface of the cell body (magenta plates in Fig. 2a, b) and that the motions of the two are the same. The raw stereocilium motion is therefore defined as the sum of the apical-surface motion relative to the camera and the stereocilium motion relative to the apical surface. We extracted the relative stereocilium motion because it dictates how MET channels are activated.

Obtaining the relative stereocilium motion from the raw motion requires knowledge of the motion of the hair-cell apical surface. However, it is difficult to visually identify the pivoting depth where the stereocilia bend or the depth where the stereocilia insert into the apical surface. To overcome this obstacle, the apical-surface motion was calculated based on stereocilium motions at multiple depths and the hair-cell-body motion measured 3–5 microns below the apical surface, with the assumption that the hair-cell apical surface moves in phase with the hair-cell body at that depth.

The coordinate systems for observing raw and relative motion are illustrated in Fig. 2a, b, respectively. To illustrate the relationship between the raw and relative stereocilium motion and the apical-surface motion, a hypothetical raw stereocilium and apical-surface motion was constructed in the time domain (Fig. 2c). The corresponding relative stereocilium motion was calculated by simple subtraction. Figure 2d demonstrates the same relationships among the motions in the frequency domain as vectors in the complex plane at the oscillation frequency, with the apical-surface motion as the reference for zero phase. In the frequency domain, the vector subtraction of the apical-surface motion (magenta arrow) from the raw stereocilium motion (solid blue arrow) yields the relative stereocilium motion (empty blue arrow).

An example stereocilium was picked to illustrate stereocilium motion in the radial direction at multiple depths (colored arrows in Fig. 2e). The measured raw radial-direction motions at these different depths are plotted in the complex plane, with the cell-body motion as the reference for phase, in Fig. 2f, g for stapes and dish stimulations, respectively. For stapes stimulation at 2 kHz, the complex plots of raw motion at different depths form a straight line (dashed magenta, *R*^2^ = 0.97). The intercept where the fitted line crosses the real axis is the calculated pivoting-point-motion vector for this stereocilium (magenta arrow in Fig. 2f). This conclusion assumes that the pivoting point and the apical surface of the cell move in phase with the cell body at a depth 3–5 microns lower. The relative-motion vectors at the corresponding depths are calculated by vector subtraction. The observation that in the complex plane the raw motions for different depths form a straight line indicates that the relative phase (*ϕ*_rel_) of the stereocilium is constant along its length. According to mechanics theory, this constant phase implies that there was little viscous loss along the stereocilium shaft, consistent with the hypothesis that the stereocilium pivots as a rigid rod.

For dish stimulation (Fig. 2g), the raw-motion vectors of the example stereocilium are roughly the same at different depths and are clustered around the real axis. As a result, the calculated apical-surface motion is also approximately the same, with the relative motions being close to zero. Note that, with such small relative motions, the phase becomes irrelevant. The above observation shows that the control experiments produced the expected outcome that the stereocilia, apical surface, and cell body all move together. This expected result together with the observation that, for stapes stimulation, the raw-motion vectors at different depths fall on one line, support the conclusion that the image-processing algorithm yields consistent results at different depths and that the detected motions are not influenced by variations in imaging conditions at different depths.

The calculation of the pivoting-point motion of each stereocilium was done in the same way for all stereocilia in the same bundle, and the mean of the results was taken as the apical-surface motion of the cell. See Fig. S3 for the details of this calculation and for a test of the assumption that the apical surface moves in phase with the cell body at a depth 3–5 microns below.

With the apical-surface motion calculated, the relative motions of individual stereocilia were obtained by vector subtraction. The results for the example bundle are shown in Fig. 2h–j. The relative stereocilium motions are mostly in the radial direction for stapes stimulation (Fig. 2h, lower panel). Summaries of the mean magnitudes in the longitudinal direction for raw and relative motions are shown in Fig. S4. The relative stereocilium motion in the radial direction is more prominent than in the longitudinal direction (Fig. S4e–j), and most of the longitudinal relative motions are <10 nm (Fig. S4f). We also obtained the magnitude and phase along the direction of maximum magnitude for each stereocilium. The inter-stereocilium differences in motion along the maximum-magnitude direction are similar to the results in the radial direction (not shown). Although the relative motion in the longitudinal direction may be physiologically important, for the sake of simplicity and fewer layers of data processing, we focus our discussion on the radial direction for relative stereocilium motions.

There are four relationships we study in the following sections: (1) how the inter-stereocilium differences relate to the mean stereocilium motion (i.e., the bundle motion), (2) how the bundle motion relates to the apical-surface motion, (3) how the pivoting of individual stereocilia relates to the apical-surface motion, and (4) how the apical-surface and cell-body motions relate to the motion of the stimulus probe. In the next section, we continue to focus on inter-stereocilium differences, thus the first relationship. In the two sections following the next, we focus on the bundle motion and stereocilium pivot angle, thus the second and third relationships. The fourth relationship is presented in Supplementary Information Fig. S5.

### Comparison of inter-stereocilium differences for 2- and 3-kHz stapes stimulation

**Fig. 3:**
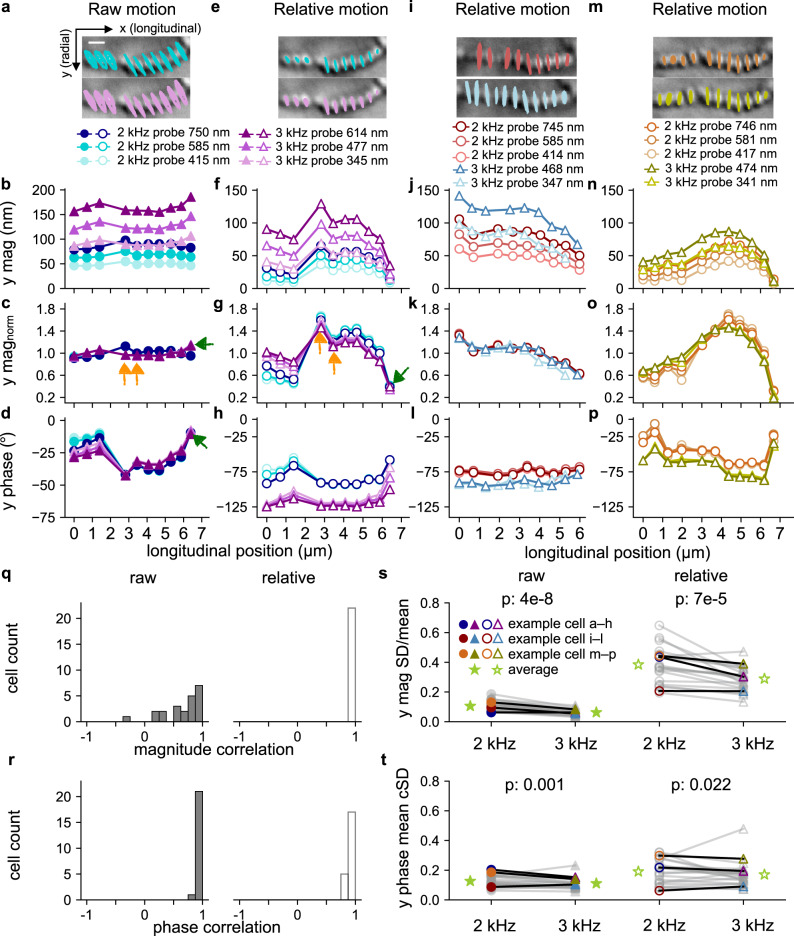
Comparing raw and relative motion under stapes stimulation at 2 and 3 kHz. **a**, **e**, **i**, **m** The motion trajectories from an example stereocilium bundle are traced on top of the bundle image (1-µm scale bar) for raw (**a**) and relative (**e**, **i**, **m**) motions, with 10× motion scaling in all panels. The 2-kHz results are in the top panels, and 3-kHz results are in the bottom panels. The same cell appears in both **a** and **e**. **b**, **f**, **j**, **n** The corresponding stereocilium-motion magnitudes in the radial (*y*) direction are plotted as functions of longitudinal position for the corresponding raw and relative motions. **c**, **g**, **k**, **o** The stereocilium magnitudes in the radial direction, normalized by the radial bundle magnitude (i.e., the mean of the individual stereocilia), are plotted for the example bundles with respect to the longitudinal position. **d**, **h**, **l**, **p** The corresponding phases of the radial stereocilium motions are also plotted against the longitudinal position. The green arrows in **c**, **d**, **g** point to a stereocilium with large raw motion (**c**) but small relative motion (**g**) and a phase close to that of the apical surface (**d**). The orange arrows in **c**, **g** point to two example stereocilia whose raw-motion magnitudes are smaller than some others (**c**), but whose relative-motion magnitudes are larger (**g**). **q**, **r** Histograms show the correlation between the 2- and 3-kHz stereocilium motion patterns for all 23 hair bundles, in terms of the normalized magnitude in **q** and phase in **r**, for raw (left) and relative (right) motions. For each cell, the normalized magnitudes and phases were averaged across input levels for each frequency. **s**, **t** The standard deviation (SD) of the inter-stereocilium magnitudes, normalized by the mean magnitude in the radial direction in **s**, and the phase SD averaged across levels in **t**, are compared pairwise between the 2- and 3-kHz stapes stimulations for all cells, with the average value of each group shown as a green star.

Comparisons between 2- and 3-kHz stapes stimulation are presented in Fig. 3. Figure 3a–h show one example bundle with its raw motions on the left and relative motions on the right, while Fig. 3i–l and Fig. 3m–p show two other example bundles, respectively. When normalized to the mean displacement magnitude of the stereocilia within a bundle, the normalized magnitudes of each stereocilium for the same frequency almost overlap across stimulation levels, both for the raw (Fig. 3c) and relative motion (Fig. 3g, k, o). This indicates that the motion magnitude of each stereocilium scales linearly with the mean magnitude of the stereocilia in the bundle as the stimulus level varies. The phase of each stereocilium is also largely consistent across stimulation levels for each frequency (Fig. 3d, h, l, p).

The relative-motion magnitudes do not shift uniformly from the raw-motion magnitudes for all stereocilia in the same bundle after the subtraction of the common apical-surface motion for these stereocilia. This is seen by the different magnitude patterns between the raw (solid purple triangles; Fig. 3c) and relative motions (open purple triangles; Fig. 3g) for 3-kHz stimulation. This is due to the differences in phase among the stereocilia within the bundle (Fig. 3d). One prominent example of this phenomenon is the stereocilium on the far right (Fig. 3c, g, green arrows), which has the largest raw magnitude among the stereocilia but the smallest relative magnitude. This is because the raw motion of this stereocilium is dominated by the apical-surface motion. After subtracting the nearly in-phase (<10°; Fig. 3d, green arrow) apical-surface motion, the relative motion of this stereocilium is very small. Due to the phase differences, some stereocilia appear to not move much in the raw motion but actually move considerably relative to the apical surface when compared to other stereocilia (Fig. 3c, g, orange arrows). Thus, the pattern of how each stereocilium moves relative to other stereocilia can be different between raw and relative motions.

The motion pattern is preserved across the two frequencies for relative motion (Fig. 3g, k, o) but not always for raw motion (Fig. 3c). To demonstrate motion-pattern correlation between 2 and 3 kHz, the Pearson’s correlation coefficient of the two frequencies’ normalized magnitudes and the two frequencies’ phases (averaged across input levels) were calculated for all cells (Fig. 3q–r). From the histogram in Fig. 3q, the magnitude patterns between the two frequencies for relative motion are highly correlated for all cells (averaging 0.95) but are less strongly correlated for raw motion (averaging 0.65). For the phase patterns, the two frequencies are highly correlated for both raw and relative motion (Fig. 3r). Thus, whatever pattern of motion a bundle exhibits, it maintains this pattern across input levels and the two frequencies tested.

The relative-motion patterns for all 23 bundles from 15 cochleae are presented in Fig. S6. The heterogeneity in the hair-bundle shapes and motion patterns is surprising. More detailed studies are needed to clarify the relationship between a hair bundle’s shape and its motion patterns. One preliminary observation is that the stereocilia at the edge(s) of individual hair bundles tend to move less when they overlap with the neighboring bundle (Fig. S6d, f–i, n, p, w).

To compare the degree of the inter-stereocilium motion differences between 2 and 3 kHz for all 23 bundles from 15 cochleae (Fig. S6), the standard deviation (SD) of the stereocilium magnitudes normalized by the mean magnitude of the bundle stereocilia (SD/mean) and the mean circular SD (cSD) of the phases are compared pairwise in Fig. 3s, t, respectively, with the raw-motion results on the left and relative-motion results on the right. The SD/mean magnitude at 3 kHz is smaller than at 2 kHz for both the raw and relative motion, and a smaller mean cSD of the phase at 3 kHz than at 2 kHz is more apparent for the raw motion (paired *t* test *p* < 0.001) than for the relative motion (paired *t* test *p* = 0.022). These results indicate that the stereocilia within a bundle move more similarly to one another in both magnitude and phase at 3 kHz than at 2 kHz.

### Bundle motion in relationship to apical-surface motion

The bundle motion is defined as the average stereocilium motion within one bundle near the top of the first-row stereocilia. In Fig. 3b, f, g, n, we observe that the stereocilia are more responsive to 3-kHz than to 2-kHz probe stimulation. At the same time, the apical-surface motion is also more responsive to the probe motion at 3 kHz than at 2 kHz (Fig. S5a, b). The larger apical-surface motion, thus larger OoC motion, at 3 kHz is expected for two reasons: (1) the cochlear input pressure is proportional to the stapes velocity^21^ and thus is 1.5× larger at 3 kHz than at 2 kHz for the same displacement magnitude; and (2) at this location, based on the passive properties of the BM and the fact that the 20-kHz CF of the imaging location is higher than the stimulation frequencies, the BM and thus the OoC is expected to be more responsive to a stimulation at 3 kHz than at 2 kHz (at a fixed location in a passive cochlea, the magnitude of BM vibration grows with frequency, peaks slightly below the CF of a live cochlea, and then drops quickly as the frequency continues to rise^1^). The bundle motion is stimulated by the OoC vibration, which is part of the traveling wave caused by the probe-induced stapes stimulation. Here we focus on the relationship between bundle motion and apical-surface motion, while the relationship between apical-surface motion, an indicator of OoC motion, and probe motion, indicating stapes motion, is studied in Fig. S5.

**Fig. 4:**
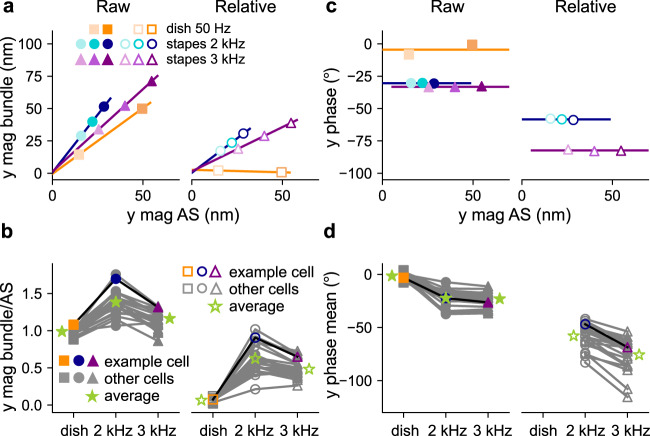
Bundle vs. apical-surface motion. The bundle motion is defined as the average motion of the tallest-row stereocilia within a bundle, as measured near the upper tips of the stereocilia. **a** The radial (*y*) bundle magnitudes are plotted against the radial apical-surface (AS) magnitude for an example bundle. In **a**–**d**, the raw bundle motion is shown on the left and the relative bundle motion is shown on the right. **b** The ratios of the bundle magnitude and the corresponding AS magnitude in the radial direction are compared among the three stimulation conditions (50-Hz dish, 2-kHz stapes, and 3-kHz stapes) for all bundles, with the average value of each group shown as a green star. **c** For the example bundle, the radial bundle phase is plotted against the radial AS magnitude, with the bundle phase calculated relative to the AS motion in each case. In **c**, **d**, the phases of the relative motion for dish stimulation are omitted because the relative motion of the bundle is so small that the confidence in the phase measurement is low. **d** The mean radial bundle phase (across input levels) is compared among the three stimulation conditions for all bundles, with the average value of each group shown as a green star.

For each stapes-stimulation case, the bundle-motion magnitude grows linearly with the apical-surface-motion magnitude, while the phase relationships stay the same (see example in Fig. 4a, c). For the control dish-stimulation experiment, the raw motion follows the apical-surface motion, i.e., they have the same magnitude (average magnitude ratio = 0.99 ± 0.04; Fig. 4b) and a close-to-zero phase difference (−1.5° ± 3°; Fig. 4d) across all cells. As a result, the relative motion is close to zero at all levels (magnitude ratio = 0.07 ± 0.04; Fig. 4b), which is expected for rigid-body motion of the whole cochlea. For stapes stimulation (Fig. 4b), the raw-motion magnitudes (Fig. 4b left panel) are greater than the apical-surface magnitude (ratios of 1.39 ± 0.16 and 1.15 ± 0.12 for 2 and 3 kHz, respectively), and the relative-motion magnitudes (Fig. 4b right panel) are 63 ± 16% and 48 ± 12% of the apical-surface magnitude for 2 and 3 kHz, respectively (paired *t* test *p* < 0.001). In terms of the phase relationships (Fig. 4d), the raw motions lag behind the apical-surface motion (left panel) by 22 ± 8° and 23 ± 7° for 2- and 3-kHz stimulation, respectively (paired *t* test *p* = 0.19). The phase lags are greater for relative motion (right panel), measuring 58 ± 11° and 76 ± 15° for the 2- and 3-kHz stimulations, respectively (paired *t* test *p* < 0.001).

In conclusion, although the bundle moves more at 3 kHz for a given probe (approximately stapes) stimulation (Fig. 3b, f, g, n), the bundle motion is greater at 2 kHz for a given apical-surface stimulation (paired *t* test *p* < 0.001, Fig. 4b).

### Pivoting of individual stereocilia and stereocilium bundles

**Fig. 5:**
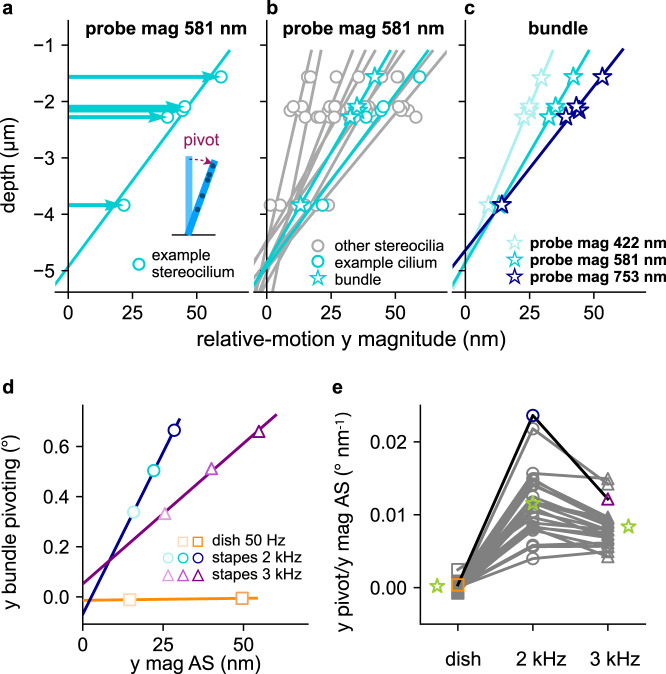
Rigid-rod-like pivoting of individual stereocilia and bundle pivoting. **a**–**c** The pivoting results are shown for 2-kHz-stapes stimulations. Note that the scales of the horizontal and vertical axes are different. **a** The radial (*y*) relative-motion magnitudes at different depths (circles) are shown for an example stereocilium, with a best-fit line shown to indicate the stereocilium. The horizontal arrows emphasize the greater magnitude of the stereocilium motion as one moves away from the pivoting point toward the tip. The inset shows a cartoon illustration of a stereocilium pivoting. **b** The results from **a** are replicated and shown alongside the results from the other individual stereocilia in the bundle (gray circles and lines), and the average for the whole bundle (stars), all from the same measurement and therefore with the same input level. **c** The radial (*y*) relative-motion results for the whole bundle (averaged across stereocilia) are shown for three different input levels. **d** The radial bundle pivoting angles are plotted against the radial AS magnitude for the example bundle. **e** The radial pivoting angle, normalized by the radial AS magnitude, is compared among the three stimulation conditions for all bundles, with the green stars representing the average value of each group.

By knowing the depth of each measurement and the relative-motion magnitudes at each depth (“Methods”), we are able to calculate the pivoting angle of individual stereocilia and the bundle (Fig. 5). It is useful to quantify the stereocilium motion using a pivoting angle, which, unlike the displacement magnitude, does not vary with the depth along the stereocilium. The results of individual stereocilia pivoting within an example bundle at one stimulation level are shown in Fig. 5a, b. Significant inter-stereocilium differences are observed. The inter-stereocilium differences in pivoting angle reflect the inter-stereocilium differences in relative-motion magnitude in the radial direction. Bundle pivoting was calculated using the average motion of the stereocilia at multiple depths (Fig. 5b, c). The magnitudes of the bundle motion at different depths appear on the same line in the depth–magnitude plot, showing that the stereocilia rotate like rigid rods (Fig. 5c,*R*^2^ = 0.997, 0.997, and 0.993 for the respective increasing stimulation levels used.). The average *R*^2^ values for all bundles of all measurements (0.97 ± 0.04) indicate that they pivot as rigid rods.

The pivoting angle of the bundle grows linearly with apical-surface magnitude for stapes stimulation, whereas for dish stimulation the pivoting angle remains near zero (Fig. 5d). The average pivoting angles per nm of apical-surface magnitude are 0.012 ± 0.004° and 0.008 ± 0.002° for the 2- and 3-kHz stimulations, respectively (paired *t* test *p* < 0.001, Fig. 5e). In the longitudinal direction, the relative bundle motion is close to zero for all bundles (Fig. S7).

### Pivoting of the tunnel of Corti and coupling of longitudinal- and radial-direction motions in the OoC

**Fig. 6:**
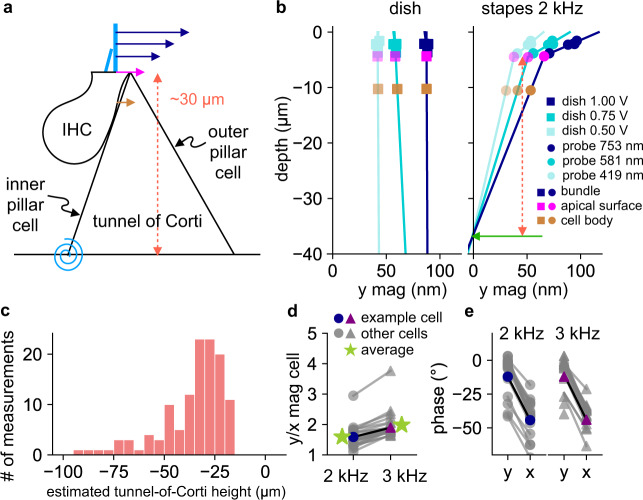
Pivoting motion of the tunnel of Corti and the coupling of longitudinal- and radial-direction motions in the OoC. **a** An IHC is drawn in relation to the triangular tunnel of Corti in a radial cross section. The motion of the IHC cell body is indicated by a brown arrow, the stereocilium motions at different depths by blue arrows, and the calculated apical-surface motion by a magenta arrow. The hypothesized point of rotation of the tunnel of Corti is indicated by the blue rotational spring at the foot of the inner pillar cell. The vertical distance between the apical surface and the base of the tunnel of Corti (the height of the tunnel of Corti) is indicated by the vertical dashed line. **b** The measurement depth is plotted against the raw motion magnitude in the radial direction for the bundle (blue), apical surface (magenta), and cell body (brown), for three different stimulation levels, with dish stimulation shown on the left and 2-kHz stapes stimulation shown on the right. The extrapolated depth of the base of the tunnel of Corti for stapes stimulation is indicated by the green arrow, with the estimated tunnel-of-Corti height indicated by the vertical dashed line. **c** The extrapolated depths of the base of the tunnel of Corti (the estimated tunnel-of-Corti height) are plotted as a histogram for all stapes-stimulation runs. **d** For all cells, the cell-body motion magnitude in the radial (*y*) direction, normalized by the corresponding longitudinal (*x*) cell-body motion magnitude, is compared between the 2- and 3-kHz stapes stimulations (*p* < 0.001). The green stars represent the average value of each group. **e** Phase comparisons between the radial (*y*) and longitudinal (*x*) motions are shown for all cells, for 2 kHz on the left and 3 kHz on the right (*p* < 0.001 for both frequencies).

Lastly, to observe the IHC motion at different depths, we compare the calculated apical-surface motion against the measured cell-body motion, which was obtained a few microns below the apical surface of the cell body (Fig. 6). For stapes stimulation, cell-body motion is consistently smaller than the apical-surface motion, whereas for dish stimulation they are about the same (Fig. 6b), which again implies rigid-body translation of the OoC during dish stimulation. Moreover, for stapes stimulation, the differences in magnitude and depth between the apical surface and cell body together indicate the pivoting angle of the IHC itself. The extrapolated location where the motion magnitude is zero indicates the point of rotation (Fig. 6b green arrow). For the example cell in Fig. 6b, this extrapolated point of rotation is 32.0, 32.3, and 32.5 μm below the apical surface for the three 2-kHz stapes-stimulation runs (pink dashed line in Fig. 6b). The histogram of the extrapolated point of rotation of all runs from all cells is shown in Fig. 6c. Most (64%) of the point-of-rotation depths lay in the range of 20–40 μm below the apical surface, with a 36.0-μm depth as the histogram average. These estimates are consistent with the height of the tunnel of Corti, measured at ~35 μm^22^, thereby supporting the idea that the tunnel of Corti rotates around the bottom of the inner pillar cell^23,24^.

It is hypothesized that the structural longitudinal coupling within the OoC, facilitated by the longitudinally inclined OHCs and phalangeal processes, is essential for the propagation of the cochlear traveling wave and the mechanisms of active amplification^25–28^. In our experiments with a passive cochlea, we observed systematic and significant longitudinal motions of the IHC apical surfaces and inner pillar cells in response to stapes stimulation, accompanying their motions in the radial direction (Fig. S5). The average ratios of radial over longitudinal motion are 1.59 ± 0.36 and 1.98 ± 0.45 for the 2- and 3-kHz stapes stimulations, respectively (paired *t* test *p* < 0.001; Fig. 6d). Moreover, the motions in the longitudinal direction are delayed more than those in the radial direction, by 33 ± 7° and 34 ± 5° for the 2- and 3-kHz stimulations, respectively (Fig. 6e). These results for the passive cochlea show that the motions in the two directions are not independent of each other near the IHC body and provide the first in situ quantitative measurements in mice of the coupling of motions between the radial and longitudinal directions in the OoC due to its structure^29–32^.

## Discussion

In situ monitoring of IHC stereocilium and cell-body motion in response to near-natural stimuli reveals how stereocilia rotate and move relative to one another within the hair bundle, how the hair bundle moves relative to the cell’s apical surface, and how the tunnel of Corti moves at two frequencies ~3–3.5 octaves below the CF. By observing the phase and magnitude at different depths we find that, relative to the apical surface, the stereocilia in the tallest row rotate as rigid rods. We show that, within the IHC hair bundle, the motions of individual stereocilia have different magnitudes and phases. The pattern of the differences between the individual stereocilia within a given bundle is highly consistent across stimulus levels and the two frequencies tested. And finally, by extrapolating the motions of the apical surface and cell body, we demonstrate that, for the mouse, the tunnel of Corti rotates around the foot of the inner pillar cell.

The observed within-bundle inter-stereocilium differences in relative motion are large. For example, at the highest stimulation level in Fig. 2j, three stereocilia moved <10 nm in magnitude, while a few moved >50 nm. The differences in phase are also significant, with the largest inter-stereocilium differences being >50° in one bundle (Figs. 2j and 3h). Since each stereocilium’s motion activates/deactivates only a few MET channels, these inter-stereocilium differences influence the overall ensemble MET current. From in vitro experiments^33,34^, the activation time of MET channels is extremely fast, thus the phase differences observed in the present experiments imply that the MET channels within a bundle will be activated at different times in one cycle of stimulation, decreasing the ensemble current due to the difference in timing between stereocilia motion. The lack of coherence among the stereocilia of one bundle, in both magnitude and phase, is unique to mammalian hair bundles, potentially offering a unique filtering opportunity, i.e., potentially attenuating the ensemble MET current below CF by virtue of the desynchrony of channel gating. Such a filtering opportunity is not present in lower-frequency hair bundles of non-mammalian species whose stereocilia are tightly coupled^35–39^. The mechanisms underlying the occurrence of these differences in motion and the exact physiological function of these differences are yet to be determined.

There are multiple hypothesized modes through which IHC stereocilia are thought to be stimulated by the vibration of the OoC and TM. These modes have different implications on inter-stereocilium motion differences for in vivo or in situ measurements with an intact TM. A classic mode, hypothesized by ter Kuile back in 1900 and still widely believed^9,40–42^, is that IHC stereocilia are moved by the differential shear motion between the TM and the stiff reticular lamina (RL) that connects the apical surfaces of the IHCs and OHCs. For this classic mode, the IHC stereocilia would likely move uniformly, despite the reported lack of coherence in vitro^15–17^, due to the uniform viscous boundary layer in the subtectorial space between the TM and the RL, especially between the tips of the tallest IHC stereocilia and the TM^9,42^. The thin fluid layer close to the surface of a solid where fluid flows by is termed a viscous boundary layer, because fluid tends to stick to or follow the surface of the solid due to viscosity effects within this layer. In the case of the subtectorial space, the solids that fluids interact with include the TM, RL, and stereocilia. It is calculated that the entire subtectorial space, ~6 μm high at the measuring location, would be within the boundary layer based on the viscosity of water (which is close to the viscosity of endolymph and perilymph) and the stimulation frequency^9,43^. In the even smaller space between the tallest-row stereocilia and the TM, the fluid and the solids would tend to move together, again due to the fluid viscosity, if the gap height remains the same throughout the motion.

Recently, alternate stimulation mechanisms for the IHC stereocilia that involve subtectorial-space fluid flow due to height changes of the space between the TM and stereocilia have been hypothesized^44–47^. For most of these hypotheses^44,47^, the change of height is small, merely reflecting the height change due to hair-bundle deflection, and the change is uniform for IHCs and OHCs. With those hypotheses, the IHC stereocilia are still predicted to move uniformly due to the effect of the viscous boundary layer since the change of height is small. However, a recent hypothesis^45,46^ predicted an opening and closing of the subtectorial space at the end near the IHC bundles, while maintaining a minimal change of the height in the OHC region. This opening and closing is facilitated by the rotation of the TM and OoC, both hinged near the inner spiral lamina (Fig. 1a). Opening and closing at the IHC end would force fluid in and out of the subtectorial space from the larger fluid space of the inner sulcus next to the IHCs (Fig. 1b). Here the viscous boundary layer would have a smaller effect on the IHC stereocilia, making it more likely that the IHC stereocilia could move more non-uniformly. The present finding of significant inter-stereocilium variance in magnitude and phase suggests a limited effect of the viscous boundary layer, and so is most consistent with the model suggesting opening and closing of the subtectorial space near the IHC bundles.

Even more recently, there is one study suggesting that the IHC stereocilia are embedded in the TM^14^. Our data neither support nor refute this possibility. The impact of embedding the IHC stereocilia into the TM is dependent on the mechanical properties associated with both the stereocilia and TM and thus requires further investigation to evaluate.

Lastly, each of the hypothesized stimulation modes would result in a different relationship between apical-surface and bundle motion. For example, with the classical shearing mode, which assumes negligible TM motion, the stereocilium displacements would have a 90° phase lag relative to the apical surface, i.e., they would be in-phase with the velocity of the RL^9,48–50^. Three pieces of data contradict this classical hypothesis. First, previous works show significant TM motion^51^. Second, the current findings concerning the phase difference between the IHC bundle and apical surface (−58 ± 11° for 2 kHz and −76 ± 15° for 3 kHz; Fig. 4d) provide further evidence that there are other stimulation modes in addition to the classical shearing mode between the TM and RL. Third, with the classical shearing mode, the raw bundle-motion magnitude would not be larger than the apical-surface motion. The greater magnitude of the raw bundle motion as compared to the apical-surface motion presented here also suggests an opening and closing of the subtectorial space near the IHC bundle, with the resulting fluid motion from or towards the inner sulcus contributing to the IHC-bundle stimulation at the frequencies tested. This is consistent with the conclusion drawn from interpreting the non-uniform motions of individual stereocilia.

Previous findings in the guinea-pig apex (CF 150–220 Hz with 200 Hz stimulation)^52^ show that the IHC bundle lags the RL by 118–155° (after converting to the same reference direction as the present work), whereas our present work on the mouse mid-turn region shows about half the lag as the previous finding. With the caveat that the previous experimental set-up was different from the current one, this comparison suggests that there could be significant variations in IHC-bundle motions across different species and/or across different cochlear locations and stimulation frequencies.

In conclusion, the present work shows that mouse IHCs display non-uniform and asynchronous within-bundle stereocilium motions for in situ preparations with an intact OoC and TM under close-to-natural stapes stimulation. This provides a unique filtering opportunity as described for mammalian IHC bundles that is not available to their non-mammalian counterparts. Moreover, the magnitude of the raw IHC-bundle motion is greater than the motion of the apical surface, which suggests that the dominant stimulation mechanism of the IHC bundles at the frequencies tested is likely fluid flowing into (out of) the subtectorial space from (to) the inner spiral sulcus near the IHC bundles due to changes in the height of the subtectorial space. Further experimental and modeling studies are needed to confirm this conclusion. We also confirmed that a stereocilium rotates like a rigid rod and that the OoC rotates around the foot of the inner pillar cell.

## Methods

### Tissue preparation

Mice of either sex at a postnatal age of 20–21 days were anesthetized (isoflurane) and decapitated using methods approved by the Stanford University Administrative Panel on Laboratory Animal Care. The left inner ear was excised, with the semicircular canals, cochlea, and stapes remaining intact (Fig. 1a, d), and then secured on top of a cover glass using a support made of light-curing dental cement (Prime-Dent Flowable). The support allowed the cochlea to be oriented such that the stereocilium bundles of the IHCs are approximately perpendicular to the imaging plane. Two holes of similar size (∼300–400 × 300–400 μm) were opened halfway along the length of the cochlea from the ST and SV sides (Fig. 1a) to allow a straight path from the light source through the condenser, OoC, and objective lens to the high-speed camera (Phantom Miro M320S). The imaging location was constrained by our ability to create an imaging path without damaging the overall structure of the cochlea. To ensure enough light was available for high-speed imaging and to limit diffraction, which degrades image quality, the RM and part of the stria vascularis were carefully removed.

During dissection, as well as imaging, the cochlea was immersed in artificial perilymph at room temperature (21–24 °C). The artificial perilymph was composed of 130.2 mM NaCl, 2.8 mM KCl, 10 mM CaCl_2_, 1 mM MgCl_2_, 10 mM HEPES, 6 mM glucose, 4 mM ascorbate, 2 mM pyruvate, and 2 mM creatine, with a final osmolarity of 308–310 mOsm and a pH of 7.4, adjusted using 1 M NaOH. The hair-cell mechano-transduction channels were blocked by incorporating 1 mM amiloride, which increases the longevity and thus the structural integrity of the tissue. Cochlear health was judged by visually observing the structural integrity of the tissue and ensuring it remained free from blebbing, OHC swelling, Brownian motion, and OHC-bundle disarray (Fig. S8). The IHCs were visually the same before and after the experiments.

A previous study^53^ showed that switching the bathing solution from an artificial endolymph (high potassium and 20 μM Ca^2+^) to artificial perilymph (high sodium and 2 mM Ca^2+^) has a minimal effect on the TM length in both the radial and longitudinal directions and causes about a 1% decrease in TM thickness. Here, we compared the appearance and position of the TM as prepared using our version of artificial perilymph with 10 mM Ca^2+^ and 1 mM amiloride (solution 2 in Fig. S9) to a case in which the tissue was prepared and imaged using a standard version of artificial perilymph with 2 mM Ca^2+^ and no amiloride added (solution 1 in Fig. S9). The TM looked the same using either version of the solution (Fig. S9) and remained unchanged for the duration of each experiment. The TM fibers remained straight and well aligned, without indications of shrinkage or swelling. The radial length of the TM was the same in all cases (Fig. S9b, d, f), reaching beyond the hair bundles of the third row of OHCs.

We could not precisely measure the thickness of the TM. However, the 1% decrease of thickness reported in the literature^53^ amounts to a 400-nm decrease given that TM thickness is ~40 μm. This change could be manifested on the side of the TM facing the SM, or on the side of the TM facing the bundles and subtectorial space, or on both sides. If this change happens solely on the side of subtectorial space, it would result in a ~6.7% (~400 nm/6 μm) increase of the height of the subtectorial space. Note that this increase is not significant enough to remove the effect of the viscous boundary layer if it is present, as the thickness of the boundary layer is predicted to be ~9–11 μm for the 2- and 3 kHz stimulations^28,43^. Therefore, this change is not predicted to invalidate the implications of our results. Given the sensitivity of the system, the actual effect of this change can be studied in detail in a future modeling project.

### Recording and stimulation

The images were obtained through the holes in the cochlea using bright-field microscopy on an upright fixed-stage microscope (Olympus BX51WI) with an Olympus ×100 1.0-NA dipping objective (1.5 mm working distance) and an additional ×2 tube lens. Using a high-speed camera (Phantom Miro M320S) together with high-power diode illumination (460 nm wavelength, Sutter Instrument), the IHC stereocilia were imaged at 12,500 fps with a system resolution sufficient to allow visualization of individual IHC stereocilia (50 nm per pixel). The stimulation frequency was set to 1, 2, or 3 kHz. The 1 kHz data were discarded due to excessive bone motion (see later section). The frame rate could not be increased, because doing so would shorten the exposure time (78 μs per frame), thus reducing light to the camera and limiting image collection. The precise synchronization between image acquisition and stimulation was achieved by controlling the camera and stimulation using a Multifunction I/O Device (National Instruments USB-6353) through a custom-built software program called jClamp (www.scisoftco.com)^54^.

Motion of the OoC was induced by directly pushing on the stapes with a glass probe attached to a piezoelectric actuator (Thorlabs AE0505D08F, Fig. 1e, f). The probe motion was monitored by laser Doppler vibrometry (Polytec OFV-511). The stimulation amplitude on the probe ranged between 100 and 800 nm. This stimulation observed at the stapes is equivalent to an ear-canal sound pressure level (SPL) of 136–156 dB SPL^55^, whereas the stimulation level reaching the OoC in the passive postmortem cochlear preparation is equivalent to an ear-canal SPL of ~80–100 dB SPL. This drop is due to the two holes on the cochlear wall and was calculated using two independent approaches: (1) by comparing against the RL motion measured in intact mouse cochleae^56^, and (2) by calculating the pressure difference using a finite-element box model of the mouse cochlea with and without holes^11,28^. Detailed comparisons and calculations for this drop are shown in Supplementary Information Methods section “Estimating the equivalent ear-canal dB SPL observed at the OoC,” as well as Table S1 and Fig. S10. The RL motion was likely further reduced compared to the same stimulation level in vivo because of the lack of active processes, which would produce significant amplification on the RL even when the stimulation frequency is lower than the CF of the location^56,57^. Lastly, the stapes motion was not directly monitored and could potentially be different from that of the probe. The coupling between the stapes and the probe is likely not a major issue, because the measured stereocilium motions at each level were symmetrical in the positive and negative radial directions (excitatory and inhibitory directions, respectively).

The stimulation duration was 20 ms per run, but the camera recorded a total of 60 ms, with an additional 20 ms before and after the stimulation. For each experimental run, the 60-ms recording was repeated 4 times back-to-back with a 100-ms pause in-between. During each 60-ms recording phase, high-power diode illumination optimized the spatial resolution, providing the necessary intensity for high-speed imaging. During non-recording time, the high-power diode was automatically switched off, and a low-power diode (530 nm wavelength, Sutter Instrument) was used for tissue preparation and orientation.

The camera was manually rotated so that the horizontal axis of the image (Fig. 1g) aligned visually with the tangent of the longitudinal direction of the BM, and the vertical axis of the image aligned with the radial direction of the BM. During the experiments, the recordings were done at 2–4 different depths along the bundle and at a further depth a few microns beneath the apical surface where the cell boundaries of the IHC and inner pillar cell were visible (Fig. 1g lower panel). Normally, the first recorded depth was the highest possible depth where the first row of stereocilia were not blurred by the TM.

As a control, the dish-stimulation experiment was designed so that all of the stereocilia (and the cell bodies) would move together as a unit. The outer dish was clamped with two pieces of rubber, one of which was pressed by the piezoelectric dish pusher as shown in Fig. 1e. The dish was pushed at a low frequency, 50 Hz, in an attempt to induce rigid-body motion of the whole cochlea. The dish-stimulation experiments were recorded at 625 fps, with the same exposure time per frame (78 μs) as the stapes-stimulation experiments for consistency. Stapes stimulation and dish stimulation were performed back to back for each field of view. To check consistency of the stimulation across different depths, after recording at all depths of interest, the stapes- and dish-stimulation experiments were repeated at the first depth recorded (see “Exclusion criteria” below).

For each cell recorded, a 10-μm-thick *z*-stack of the bundle, consisting of 200 frames in 50-nm steps, was taken before and after the experiments to produce a 3-dimensional bundle structure. In addition, a *z*-scan of the OoC from the TM through the bottom of the OHCs was recorded before and after the experiments as a way of gauging tissue health (Fig. S8).

### Motion detection

Motion detection was performed using the TrackMate plugin of Fiji (ImageJ)^58,59^. The algorithm involves two steps: spot detection for each frame and a spot-linking step that links the same spots between frames. For the spot-detection step, TrackMate applies a Laplacian of Gaussian (LoG) filter to the image and finds the locations of the local maxima of the filtered image. Sub-pixel localization of the spots is enabled by applying a quadratic fit using the pixels around the maxima (±1 pixel in both directions). For the spot-linking step, TrackMate implements the Linear Assignment Problem method developed by Jaqaman and colleagues^59^.

A single parameter, the SD of the LoG filter, is used in the spot-detection step. This parameter is automatically optimized based on the estimated spot size that the user provides. We tried three sizes, 300, 400, and 500 nm in diameter, and found that the results (magnitude and phase of the motion) were rather insensitive to this parameter. We chose to set the SD parameter to 400 nm because doing so allowed more stereocilia to be successfully tracked in the spot-linking step based on our selection criterion (described in the next paragraph). For the spot-linking algorithm, three parameters were provided to limit the possible combinations and thus speed up the computation: the maximum linking distance, maximum gap-closing distance, and maximum number of gap frames. The gap in the last two parameters refers to the situation when an object is not detected for a few frames. For our case, because one stereocilium does not move >200 nm during maximum stimulation, we set the first two parameters to 200 nm. To ensure the quality of tracks, we set the maximum number of frames in a gap to 2.

An additional selection criterion for tracks was based on the total number of frames in gaps. Tracks were discarded if there were >10 gap frames for the 3000-frame recording (totaling 3000 frames for both stapes and dish stimulation). For the case of stereocilium motion, the computed tracks were further examined by eye to discard tracks that were not stereocilium motion. For cell-body or bone motion, all the tracks that passed the above criterion were used, and the average was used as an estimate of the cell-body or bone motion, respectively.

The validation of stereocilium sub-pixel motion measurements using bright-field high-speed imaging and a motion detection algorithm was done in our previous work (Caprara et al.^60^), where the image processing was validated by comparing the results to a classical dual photodiode motion-detection system^61^ for frequencies up to 9 kHz. For the present work, the same microscope with the same lens and same high-speed camera and software were used. However, the image-processing algorithm used in Caprara et al. was a Gaussian one-dimensional (1D) fit, which is different from what we needed and used in the present work. To demonstrate that the type of algorithm does not affect the sensitivity and precision of the measurement, we compare the results of TrackMate in one direction to that of the Gaussian 1D fit in the same direction (radial) on individual stereocilia from six randomly chosen samples collected from the present work. Details are presented in Supplementary Information section “Comparing Gaussian 1D fit and Fiji ImageJ plugin TrackMate” and Fig. S11. The results show that there is no systematic difference between the two methods. Since the Gaussian 1D fit was validated by photodiode in Caprara et al., we conclude that TrackMate is also validated.

### Magnitude, phase, and SD of the magnitude and phase

The motion-detection algorithm was applied to the recordings of the four repeats of the same protocol. The average motion of the four repeats was taken as the time-domain data for one stimulation protocol (Fig. 1h). To convert this time-domain data into frequency-domain data (magnitude and phase), the resulting 20-ms stimulation period was divided into 10 segments of equal duration. The first two segments were discarded to capture only the steady-state behavior, and a Fast Fourier Transform was applied to each of the last eight segments. The average of the magnitudes from those eight segments was then taken as the magnitude of the steady-state signal. The SD of the magnitudes across the eight segments was also computed. To obtain the phase difference between a pair of time-domain signals, the mean and cSD of the eight phase differences between the respective paired segments were obtained using a standard method in circular statistics^62^, which is outlined in the Supplementary Information section “Calculating the circular standard deviation of the phase”. Note that the cSD of phase differences is unitless and that this method was also used for calculating the mean and cSD of the phase differences across stereocilia in the same bundle and across bundles.

The cSD of the phase across the eight segments is an indicator of the confidence of the phase calculation. The larger the value, the less confident the calculation. Thus, it is useful for identifying unreliable phase measurements, which is usually due to the motion being too small. The cSD of the phase across segments grows as the motion magnitude decreases, as shown in Fig. S12. Stereocilium motions with the cSD of the phase across segments >0.01 or motion magnitude <5 nm were discarded in the calculations of the mean and cSD for the inter-stereocilium phase.

Note that the SD of the magnitude and cSD of the phase across segments only indicate the variation present across the eight segments for the motion of a single stereocilium. They describe the consistency of the steady-state motion and the image-processing algorithm during one stimulation run for a given stereocilium, but because they do not account for bias due to differences in the shape and image quality of different stereocilia within the same bundle, the dish-stimulation experiments are also needed. The SD of the magnitude across the eight segments (1.9% and 1.2% in respective longitudinal and radial directions, averaged across all traces) and cSD of the phase across the eight segments (3 × 10^−4^ and 1.5 × 10^−4^ in respective longitudinal and radial directions, averaged across all traces) turn out to be very small, aside from the reasonable exception in the phase when the motion is too small, as is explained in the previous paragraph. As these variances across segments for individual stereocilia are small compared to the differences observed across stereocilia during the dish-stimulation experiment, they are not shown in plots.

### Exclusion criterion for bone motion

The motion of the cochlear bone was recorded while stimulating the stapes at each stimulation frequency (Fig. S13) after the main experiments. During 1 kHz stimulation, the cochlear bone showed significant motion between 5 and 20 nm in the longitudinal or radial direction for 5 out of 7 specimens (Fig. S13). This indicates that the stapes stimulation at 1 kHz somehow translated into bulk motion of the cochlea instead of a simple relative motion between the stapes and fixed cochlea. This additional bone motion is undesirable because it complicates the stimulation mode by adding to and obscuring the measured motions of the bundle and apical surface. We suspect that the significant bone motion at 1 kHz was due to a resonance resulting from a particular combination of the stiffness of the stapes ligament, stiffness of the dental cement, and the traction between the cochlear bone and dental cement. Regardless of the reasons behind it, the 1-kHz data were discarded due to the excessive bone motion. For the 2- and 3-kHz stimulations, the bone motion varied between 0 and 5 nm in magnitude in both the longitudinal and radial directions, and so no cells were excluded based on bone motion.

### Exclusion criterion for repeatability

The validity of the analysis requires repeatability of the results when the same stimulation protocol is applied at different times and at different depths. To check whether this was approximately true, the measurements at the first depth were repeated after finishing the recordings at all other depths. For 32% of the preparations, very little motion was induced in the last stimulation, likely due to a shift in the engagement between the stapes and probe and/or deterioration of the structural/mechanical properties of the tissue. Measurements from those preparations were excluded.

### Determination of the recording depth

Both the stapes- and dish-stimulation recordings were made at different depths along the stereocilium bundle. The distances between these depths were not immediately known, because, although we had control of the depth of the objective lens, the tissue could drift up or down a noticeable amount over the duration of the experiment (~1–2 microns during a 30-min experiment). The relative depths, however, are needed for calculating the pivoting angles of the stereocilia. Thus, the method presented here was used to determine the relative depths of the recordings based on the *z*-stack images of the bundle taken before the experiments. Note that the 200 frames in the *z*-stack were taken within a second, thus the drift of the tissue during this time can be ignored. The basic idea of the method is to compare the first frame of each stimulation run (the target image) against the 200 frames in the *z*-stack (reference images) to find the most-similar (least-different) frame from the stack (Fig. S14a). Since the steps between frames in the z-stack are known (50 nm), the distances between the target images could thus be determined. Sub-step depth resolution is achieved using a second-order polynomial fit (Fig. S14b).

The difference between the target and reference images is calculated using the Python function register_translation()^63^ from the scikit-image package^64^, which returns the best translational shift between the two images so that the difference between the two images is minimized, as well as the difference after the best shift (to the target image). It is important to apply the best shift before calculating the difference between the target and reference images because the tissue drifts slightly between recordings. The algorithm used by register_translation() for finding the best translational shift is to perform a cross-correlation of the two images in the frequency domain and to find the shift in pixels at which the cross-correlation matrix has its maximum absolute value (indicating the greatest similarity)^63^. The difference between the best-shifted target and reference images is defined as

$${{{difference}}}=1-\frac{{{{{corr}}}}_{{{{{{\rm{max }}}}}}} * {\bar{{{{{corr}}}}}_{{{{{{\rm{max }}}}}}}}}{{{{{amp}}}}_{{{{{{\rm{img}}}}}}1} * {{{{amp}}}}_{{{{{{\rm{img}}}}}}2}},$$

where *corr*_max_ is the entry of the cross-correlation matrix that has the maximum absolute value, and *amp*_img1_ and *amp*_img2_ are the average squared intensity of the two images in the frequency domain, respectively. In addition, the two images were normalized to their own highest intensity prior to applying register_translation().

After the differences were calculated between the best-shifted target and reference images (blue dots in Fig. S14a, b), the raw differences were smoothed using a Savitzky–Golay filter (11-point window, order 2), which fits successive subsets of adjacent data points with a polynomial (pink lines in Fig. S14a–b). The sub-step resolution between *z*-stack frames was determined using the second-order polynomial fit applied onto the smoothed-differences around their minimum (±5 points).

Finally, the accuracy of this method was tested by applying it to the two *z*-stacks of the same bundle taken before and after the stimulation experiments, using the images from one stack as the target images and the others as the reference. In other words, we determined the depth of each image in the target stack in terms of the frame number (or depth) of the images in the reference stack and plotted the result against the frame number (or depth) of the target stack itself (Fig. S14c, orange line). Both stacks were acquired with a step size of 50 nm, so the slope of the calculated depth vs. known depth should be one. We know that the two stacks do not necessarily start or end at the same depth, so a shift was expected. Therefore, a straight diagonal line with slope of one (Fig. S14c, green line) was shifted up or down to best-fit the calculated depth (orange line). The deviations between the best-shifted depth (green line) and calculated depth (orange line) represent the errors of this method for this specific sample. The maximum and SD of the errors for each specimen were calculated and are shown in Fig. S14d. The specimens with a maximum error >0.5 μm were excluded from the pivot calculations.

### Reporting summary

Further information on research design is available in the Nature Research Reporting Summary linked to this article.

## Supplementary information

Supplementary Information

Reporting Summary

## Data Availability

The data sets generated during and/or analyzed during the current study are available from the corresponding author on reasonable request. Source data for figures are deposited to repository Figshare at https://figshare.com/projects/In_Situ_Motions_of_Individual_Inner-Hair-Cell_Stereocilia_from_Stapes_Stimulation_in_Adult_Mice/116343.

## Code availability

All the custom programming code for image processing, data analysis, and plotting are written in Python with packages NumPy, SciPy, pandas, scikit-image, and Matplotlib. The original scripts are available at https://github.com/RicciLab/In-Situ-Motions-of-Individual-Inner-Hair-Cell-Stereocilia.
