# Supplementary material for: In situ motions of individual inner-hair-cell stereocilia from stapes stimulation in adult mice
Source: Commun Biol. 2021 Aug 11;4:958. doi: 10.1038/s42003-021-02459-6 (PMC8357788; doi:10.1038/s42003-021-02459-6)
Supplement: Supplementary file 1 — Supplementary Information [file 42003_2021_2459_MOESM1_ESM.pdf]

In Situ Motions of Individual Inner-Hair-Cell Stereocilia from Stapes Stimulation in Adult Mice

Yanli Wang[1,2,3], Charles R. Steele[2], Sunil Puria[3,†], and Anthony J. Ricci[1,†,*]
[1]Otolaryngology-HNS, Stanford University, Stanford, CA
[2]Mechanical Engineering, Stanford University, Stanford, CA
[3]Massachusetts Eye and Ear, Harvard Medical School, Boston, MA
[†]These authors jointly supervised this work
*Correspondence to: aricci@stanford.edu

Supplementary Information Contents

Supplementary Results

1. **Comparison of raw stereocilium motions between dish and stapes stimulation**
2. **Effect of motion along the optical axis**
3. **Calculating the apical-surface motion**
4. **Inter-stereocilium differences in longitudinal motion**
5. **Apical-surface and cell-body motion as a function of the stapes-probe input**
6. **Motion patterns of all bundles**
7. **Bundle motion relative to apical-surface motion in the longitudinal direction**

Supplementary Methods

8. **Maintaining tissue integrity**
9. **Tissue integrity of the tectorial membrane**
10. **Estimating the equivalent ear-canal dB SPL observed at the OoC.**
11. **Comparing Gaussian 1D fit and Fiji ImageJ plugin TrackMate.**
12. **Calculating the circular standard deviation (cSD) of the phase**
13. **Bone motion**
14. **Determination of the recording depth**

Supplementary Results

Comparison of raw stereocilium motions between dish and stapes stimulation. Raw motion refers to the motion directly captured by the camera and extracted by the motion-detection algorithm. For the purpose of validating the motion-detection algorithm, the raw stereocilium motion in the radial (y) direction, measured near the top of the stereocilia, is compared between dish stimulation at 50 Hz and stapes stimulation at 2 kHz for an example bundle in Figure S1a–f. The dish stimulation was performed at a lower frequency in the hope of achieving quasi-static motion of the preparation without resonating with the soft tissue within the organ of Corti (OoC). The stereocilia within the same bundle moved more differently from one another in both magnitude and phase during stapes stimulation (Fig. S1d,f) than they did during dish stimulation (Fig. S1c,e). The phase difference was calculated relative to the inner hair cell (IHC) cell-body motion throughout the text unless otherwise specified. To quantify the differences in motion among the stereocilia within a bundle, we calculated the inter-stereocilium standard deviation (SD) of the magnitude and the circular standard deviation (cSD) of the phase (note that the cSD of the

phase is unitless; see “Calculating the circular standard deviation of the phase” in the SI Methods). In Figure S1g–h, the SD of the magnitude and cSD of the phase for the example bundle are plotted against the bundle magnitude, which is defined as the magnitude of the mean motion of all successfully measured stereocilia in that bundle. The inter-stereocilium SD of the motion magnitude grows linearly with the bundle magnitude for both dish and stapes stimulation (R^2 = 0.94, 0.98, and 0.79 for the dish, stapes at 2 kHz, and stapes at 3 kHz, respectively; Fig. S1g). Thus, to compare the inter-stereocilium magnitude spread between dish and stapes stimulation, the slope, i.e., magnitude SD/mean, is used (Fig. S1i). The cSD of the phase was found to be insensitive to the bundle magnitude (Fig. S1h). Thus, to compare the inter-stereocilium phase spreads, the cSDs of the phases at different stimulation levels were averaged for each stimulation method and frequency (Fig. S1j). The pairwise comparisons between the dish and stapes stimulation of the magnitude SD/mean (Fig. S1i) and the mean cSD of the phase (Fig. S1j) for all bundles indicate that the stereocilia moved significantly more differently from one another, in both magnitude and phase, with stapes stimulation (for both 2 and 3 kHz) than with dish stimulation (paired t-test p-values < 0.001 for all cases). These results indicate that the inter-stereocilium differences in motion during stapes stimulation are not artifacts of the methods used to measure and calculate the motions.

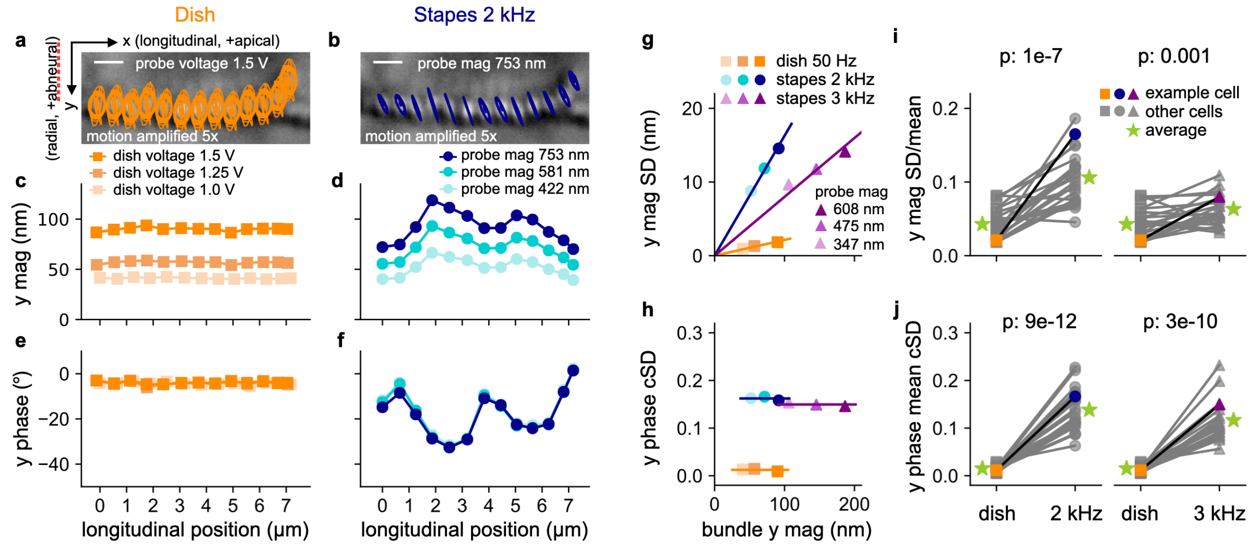

Figure S1. Comparisons of raw stereocilium motions for dish vs. stapes stimulations. a–f Stereocilium motions caused by dish stimulation at 50 Hz (left column) and stapes stimulation at 2 kHz (right column) are shown for an example bundle. The input level for dish stimulation is reported in terms of the voltage applied to the piezoelectric transducer, because the motion of the dish stimulator was not measured, whereas for stapes stimulation the input level is reported as the peak probe displacement, which was measured by the LDV. Note that the coupling between the probe and the stapes head was not monitored, and thus the two motions could differ. **a,b** The motion trajectories (scaled 5x) are traced on top of the original image for the highest input levels, with 1-µm scale bars. **c–f** The magnitudes (c,d) and phases (e,f) of the motions in the radial (y) direction of 13 individual stereocilia are plotted against the longitudinal position of each stereocilium, spanning about 7 µm (0 marks the initial position of the leftmost stereocilium). The phases are relative to the cell body throughout the text, unless otherwise specified. **g,h** The inter-stereocilium standard deviation (SD) of the magnitudes in the y-direction motion and the inter-stereocilium circular standard deviation (cSD) of the phase are plotted in g and h, respectively, as functions of the magnitude of the mean (y-direction) motion of the stereocilia in the bundle (i.e., the bundle magnitude). A higher SD or cSD indicates lower uniformity of the stereocilium motions in the hair bundle. **i** Pairwise

comparisons are shown of the magnitude SD divided by the bundle magnitude, between dish and 2-kHz-stapes stimulation on the left, and dish and 3-kHz-stapes stimulation on the right, with p-value results from paired Student's t tests shown above the plots. **j** Pairwise comparisons are shown of the phase cSD, averaged across the different input levels, between dish and 2-kHz-stapes stimulation on the left, and between dish and 3-kHz-stapes stimulation on the right, with p-values above the plots. In (i–j), the results from the example bundle in (a–f) are colored according to the stimulation type, with the other cells shown in gray, and the average across cells in each group indicated by a green star.

Effect of motion along the optical axis. Stereocilium motion in the direction perpendicular to the imaging plane (i.e., motion along the optical axis) will produce apparent motion in the imaging plane (in-plane motion) due to the inclination angles of the stereocilia relative to the imaging plane. An illustration of this phenomenon is shown in Figure S2b where the position of an example stereocilium, marked by cyan and pink dots in Fig. S2a, is plotted at different depths (50-nm step size; positions obtained from a z-stack scan and image processing). For this example, an out-of-plane motion of Δz of 2 μm would result in an in-plane motion in the radial (Δy) and longitudinal direction (Δx) of ~0.3 and ~0.02 μm, respectively. Given this, one could hypothesize that the observed inter-stereocilium motion differences during stapes stimulation might be the result of motion along the optical axis combined with variability among the inclination angles of the stereocilia. To examine this possibility, we studied whether there is a correlation between larger (or smaller) stereocilium motion and larger (or smaller) inclination angles of the stereocilia within a bundle, in both radial and longitudinal directions.

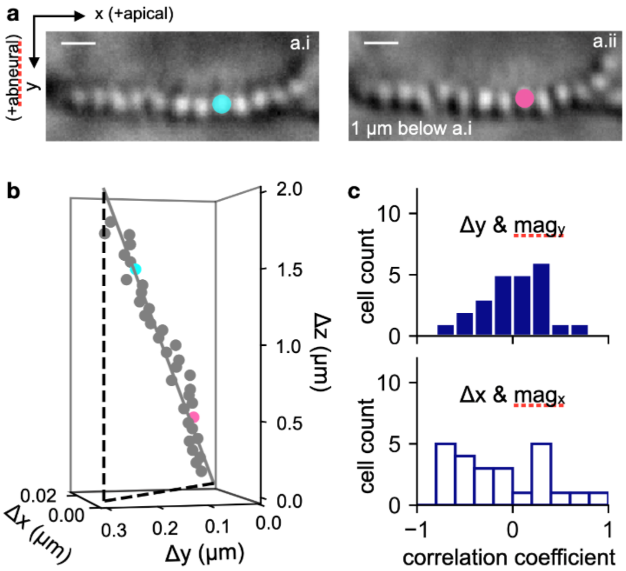

Figure S2. Gauging the potential influence of motion along the optical axis. a In these two images of different depths from the same z-stack, an example stereocilium is colored in cyan in the upper image at the first depth, and in pink in the lower image taken at a depth 1 μm below the first image. The scale bars indicate 1 μm. **b** The 3D positions of the example stereocilium are plotted, as traced through part of the z-stack, with the positions from the two images in (a) colored accordingly in cyan and pink. The inclination angle of the stereocilium shows that a motion along the optical axis Δz could produce apparent longitudinal (Δx) and radial (Δy) motion within the imaging plane. **c** The distributions of correlation coefficients are shown between Δy and mag_y (upper row), and Δx and mag_x (lower row), for the stereocilia in one bundle with stapes stimulation at 2 kHz.

First, the inclination angles of the individual stereocilia were obtained from the z-stack profile of the bundle (one example result is shown in Fig. S2b). Next, to study the possible relationships between the potential apparent in-plane motion (Δx and Δy) caused by motion along the optical axis (Δz) and the actual measured stereocilium-motion magnitude during stapes stimulation (mag_x and mag_y), the correlations between Δx and mag_x, and between Δy and mag_y were calculated. Because the correlation coefficients are independent of the specific value of Δz used, an arbitrary value of 50 nm was chosen (Fig. S2c). From the stapes-stimulation histograms (Fig. S2c, left), there is no correlation (mean correlation coefficient = 0.008) in the radial (y) direction (upper plot) between the measured motion for 2-kHz stapes stimulation and the apparent motion due to the inclination angle, and a weak negative correlation (mean correlation coefficient = – 0.127) in the longitudinal (x) direction (lower plot). The radial-direction results with stapes stimulation indicate that the inter-stereocilium differences in this direction are not related to different inclination angles among the stereocilia combined with motion along the optical axis.

Calculating the apical-surface motion. Upon stapes stimulation, each stereocilium pivots like a rigid rod. This is evident in the complex-plane plots for the stereocilium motion (Fig. S3b), in that the ends of the raw-motion vectors (blue triangles), each representing a different depth, mostly line up along a straight line. Note that the same cell-body motion is used as the phase reference for all stereocilia in that cell. With the assumption that the phase of the pivoting-point motion is the same as the cell-body motion, the intercept of each best-fit line (dashed blue) with the real axis represents the calculated magnitude of the pivoting-point motion for that stereocilium (Fig. S3b). By taking the average of all the calculated pivoting-point magnitudes (the intercepts with the real axis) from all of the stereocilia, we obtain the magnitude of the apical-surface motion. Again, the phase of the apical-surface motion is assumed to be the same as that of the pivoting-point motion and the cell-body motion about 3–5 microns below the apical surface.

Notice that there are some stereocilia that barely moved relative to the pivoting point or apical surface. The raw motions of these stereocilia are mostly composed of apical-surface motion, so their raw-motion vectors end up being close to the real axis with minimal imaginary parts. Examples of these stereocilia are those numbered 8, 9, 10, and 12 in Figure S3b, plotted in gray. The raw-motion vectors of these stereocilia are less suitable for a line fit because the points are close to each other, and thus the confidence of the fit is low. For this reason, we have excluded from the calculation of apical-surface motion those stereocilia whose largest absolute imaginary part is less than half of the median of the population of stereocilia in the same bundle. The mean magnitude of the calculated apical-surface motion from the rest of the stereocilia is taken as the apical-surface motion magnitude of the cell. The standard error (SE) divided by the mean (SE/mean) of the apical-surface motion magnitude for each cell is shown in Figure S3d.

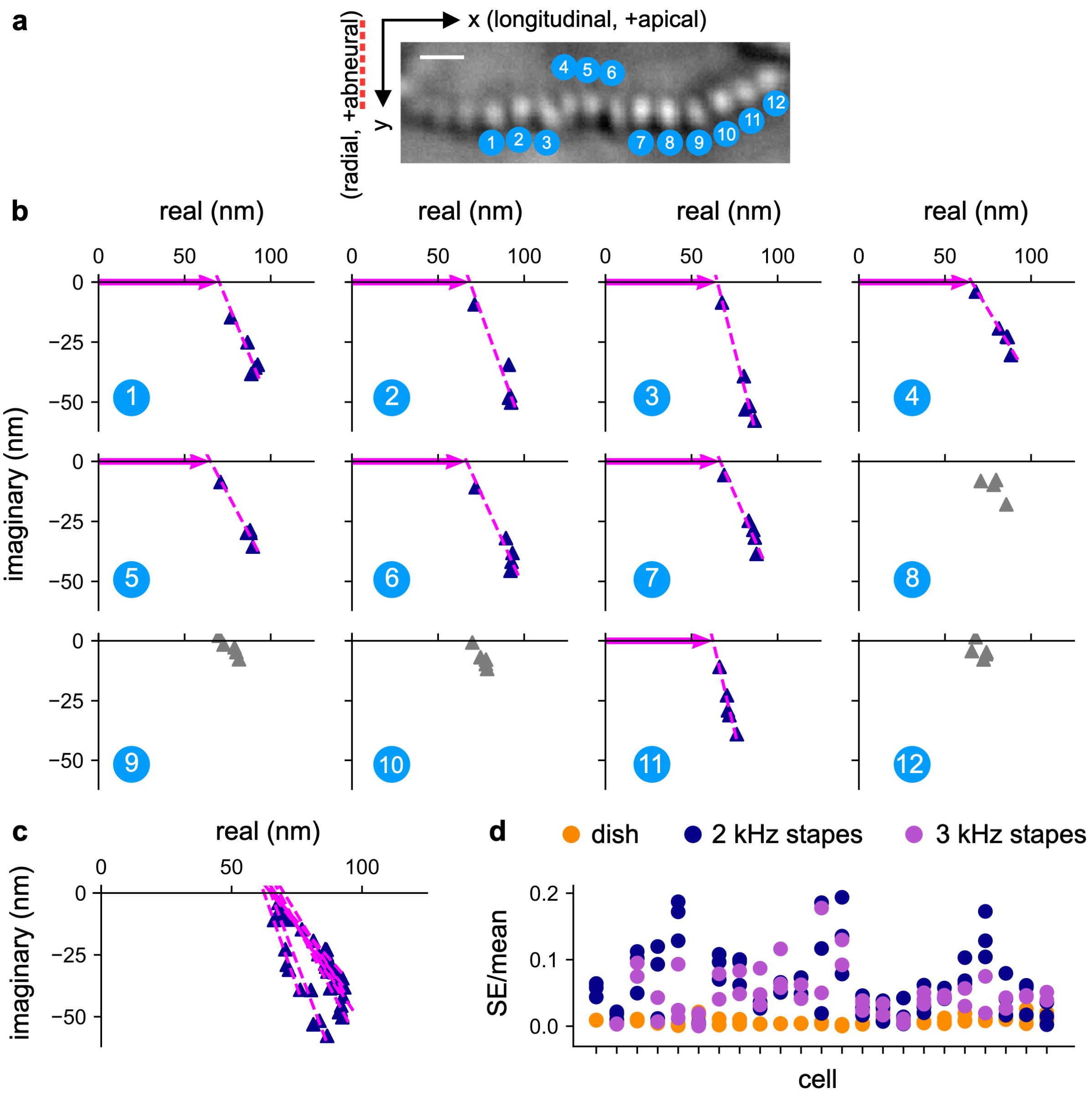

Figure S3. Calculating the apical-surface motion. a A calculation of the apical-surface motion for this example cell (with a 1-μm scale bar) is attempted based on the raw motions of the 12 numbered stereocilia. **b** The complex amplitudes of the raw motions in the radial direction of each numbered stereocilium are shown in separate panels in terms of the real and imaginary parts at each depth along the stereocilium (as in Fig. 2f). For each stereocilium, the ends of the raw-motion vectors (blue triangles) are fitted to a straight line (dashed magenta), and the intersection of this line with the real axis represents the calculated apical-surface-motion vector for that stereocilium (magenta arrow). The stereocilia whose raw-motion vectors are plotted in gray (#8, 9, 10, and 12) have maximum absolute imaginary parts that are less than half of the median of the full population of 12 stereocilia. Because of their small imaginary parts, the points are clustered around the real axis, thus making them less suitable for straight-line fitting to calculate the intercept with the real axis. For this reason, these 4 stereocilia are excluded from the calculation of apical-surface motion. **c** The raw-motion vectors for the 8 stereocilia that are not excluded are compiled onto a single plot. The mean of the calculated apical-surface motions, from the 8 stereocilia in this example, is taken as the apical-surface motion of the cell. **d** The standard error (SE) of the mean apical-surface magnitude, normalized by the same mean magnitude, is shown for each cell. Each dot represents a stimulation run color-coded according to the stimulation type and frequency.

Inter-stereocilium differences in longitudinal motion. The raw- and relative-motion results in the longitudinal (x) direction are organized in Figure S4. Similar to the comparisons made for motion in the radial (y) direction in Figure S1i,j, in Figure S4a,b the inter-stereocilium SD/mean magnitude and mean phase cSD of the raw motion are compared for the longitudinal (x) direction between dish stimulation and stapes stimulation at 2 and 3 kHz. As for the radial-direction results, the longitudinal-direction inter-stereocilium differences are greater for stapes stimulation than for dish stimulation for both magnitude and phase.

The mean magnitudes of stereocilium motion in the longitudinal direction tend to be much smaller than in the radial direction. The distributions of these two quantities are shown in Figures S4e,f and S4g,h, for raw and relative motion respectively. The distributions of the ratio of the motion in the two directions from the same run are shown in Figure S4i,j. After subtracting out the apical-surface motion, the relative stereocilium motion in the longitudinal direction (Fig. S4f) becomes much smaller than in the radial direction (Fig. S4h), with most runs falling below 10 nm in the longitudinal direction. For simplicity, therefore, the main inter-stereocilium results of the current study are focused on motion in the radial direction.

As in Figure 3k,l for the radial direction, the inter-stereocilium SD/mean magnitude and mean phase cSD for the longitudinal direction are compared between 2 and 3 kHz for both raw and relative motion in Figure S4c,d. For the raw longitudinal motion, the inter-stereocilium differences due to 3-kHz stimulation are smaller in both magnitude and phase than those due to 2-kHz stimulation, which is also the case for the radial motion. For the relative longitudinal motion, however, there is no significant difference in the inter-stereocilium variability in either magnitude or phase between the 2- and 3-kHz stimulations (right column of Figure S4c), which contrasts with the radial-motion behavior.

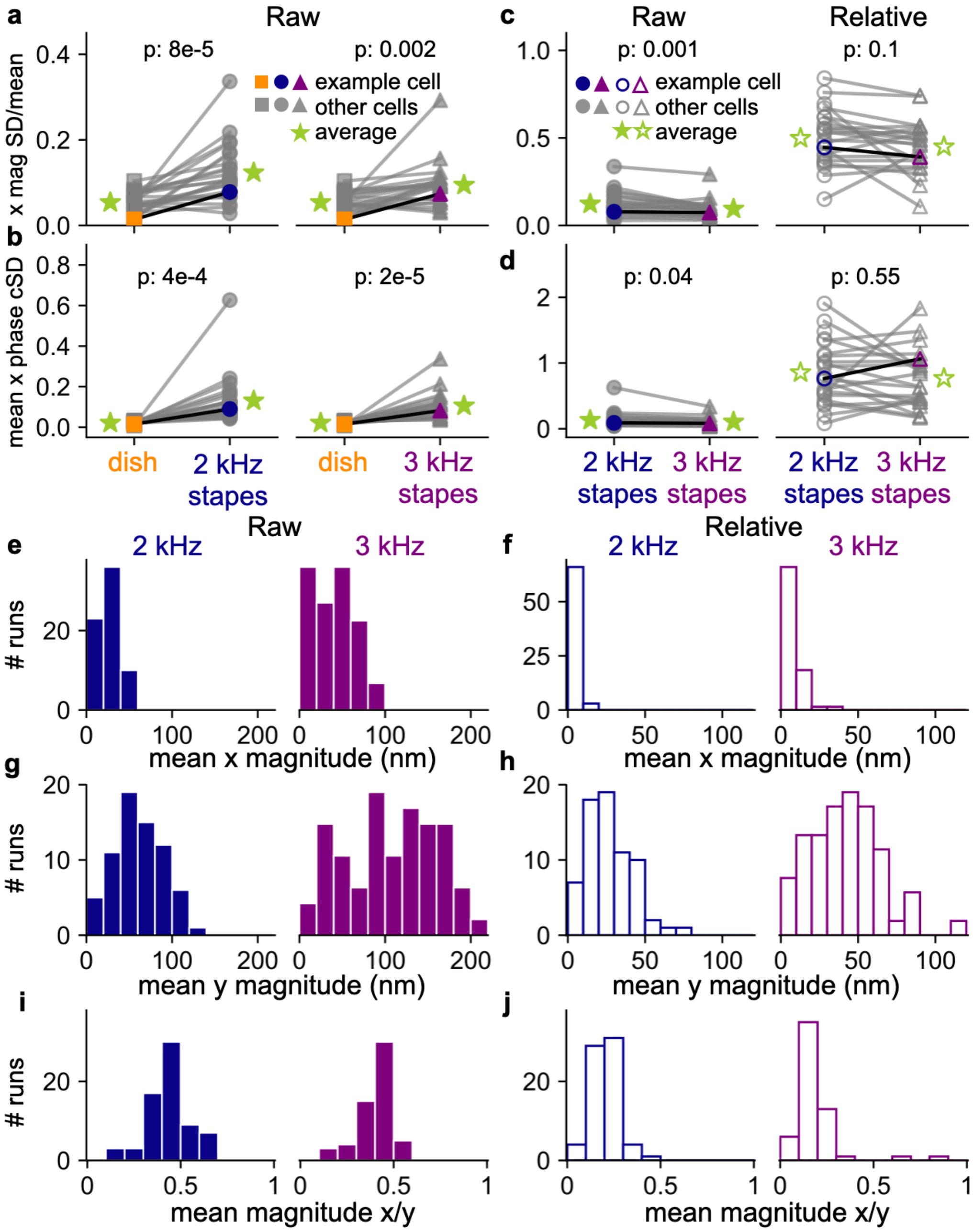

Figure S4. Inter-stereocilium differences in the longitudinal (x) direction and mean magnitudes. a The raw inter-stereocilium magnitude SD/mean results in the longitudinal (x) direction are compared between dish (left points in each sub-panel) and stapes stimulations (right points in each sub-panel) at 2 and 3 kHz. **b** The raw longitudinal inter-stereocilium cSDs of the phase relative to the cell body, averaged across input levels, are compared between dish and stapes stimulations. **c** The longitudinal inter-stereocilium SD/mean magnitudes are compared between 2- and 3-kHz stapes stimulation, for raw motion on the left and relative motion on the right. **d** The longitudinal inter-stereocilium cSDs of the phase relative to the cell body, averaged across input levels are compared between 2- and 3-kHz stapes stimulation, for raw motion on the left and relative motion on the right. **e,f** The distributions of the mean longitudinal stereocilium magnitudes are shown for the raw and relative motion in (e) and (f), respectively. **g,h** The distributions of the mean radial stereocilium magnitudes are shown for the raw and relative motion in (g) and (h), respectively. **i,j** The distributions of the longitudinal-over-radial ratios of the mean stereocilium magnitudes are shown for the raw and relative motion in (i) and (j), respectively.

Apical-surface and cell-body motion as a function of the stapes-probe input. Both the apical-surface (AS) and cell-body motions are linear with respect to the input stimulus provided by the stapes probe, in both longitudinal and radial directions (Fig. S5a and S5c). The ratios of the AS and probe magnitudes, and the cell-body and probe magnitudes are shown in Figure S5b and S5d, respectively, for all cells. Stapes stimulation at 3 kHz generated more AS and cell-body motion per unit input at the probe than did stapes stimulation at 2 kHz, in both directions ($p < 0.001$).

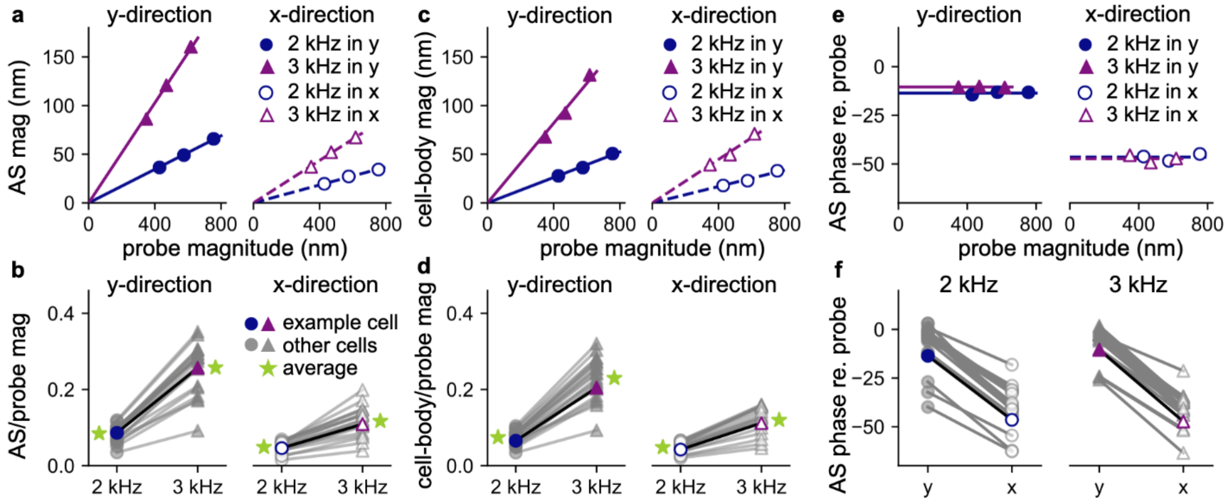

Figure S5. Apical-surface and cell-body motion vs. stapes-probe input. **a** The motion magnitude of the apical surface (AS) is plotted against that of the input probe for an example cell at 2- and 3-kHz stapes stimulation, with the radial (y) motion on the left, and longitudinal (x) motion on the right. **b** Ratios of the AS and probe magnitudes for all cells, in both directions, are compared across the two stapes-stimulation frequencies. **c** Here, the motion magnitude of the cell body is plotted against that of the input probe, similarly to (a). **d** Ratios of the cell-body and probe magnitudes for all cells are compared, similarly to (b). **e** The phase differences between the AS and input probe are plotted with respect to the magnitude of the probe, in terms of both input frequencies and directions, for the example cell. **f** The AS phase relative to the probe, averaged across input levels, is compared between the two directions at both input frequencies. No significant differences in the phase results are seen between the 2- and 3-kHz stimulations.

The phase differences between AS and probe motion are shown in Figure S5e, for the example cell in Figure 5. These phase delays are independent of the stimulation level, so the averages across levels (horizontal lines) are compared between the different cases. The difference between the 2- and 3-kHz phase results is insignificant for both directions ($p = 0.68$ and 0.19 for the longitudinal and radial direction, respectively). The phase delays across all cells are compared between the radial (y) and longitudinal (x) directions in Figure S5f. The longitudinal motion is delayed more than the radial motion, by 33 ± 7° and 34 ± 5° for 2- and 3-kHz stimulations, respectively.

Motion patterns for all bundles. The relative-motion magnitudes, normalized by the mean magnitude of the stereocilia in one bundle, and relative-motion phases are shown in Figure S6 for the stereocilia of all 23 bundles from 15 cochleae. It is observed that the stereocilia near the edge(s) of a bundle tended to move less when there was overlap with a neighboring bundle (Fig. S6d,f,h,i,j,k,l,r). Other than this observation, the motion pattern of each bundle is somewhat unique, as is the shape of the bundle itself.

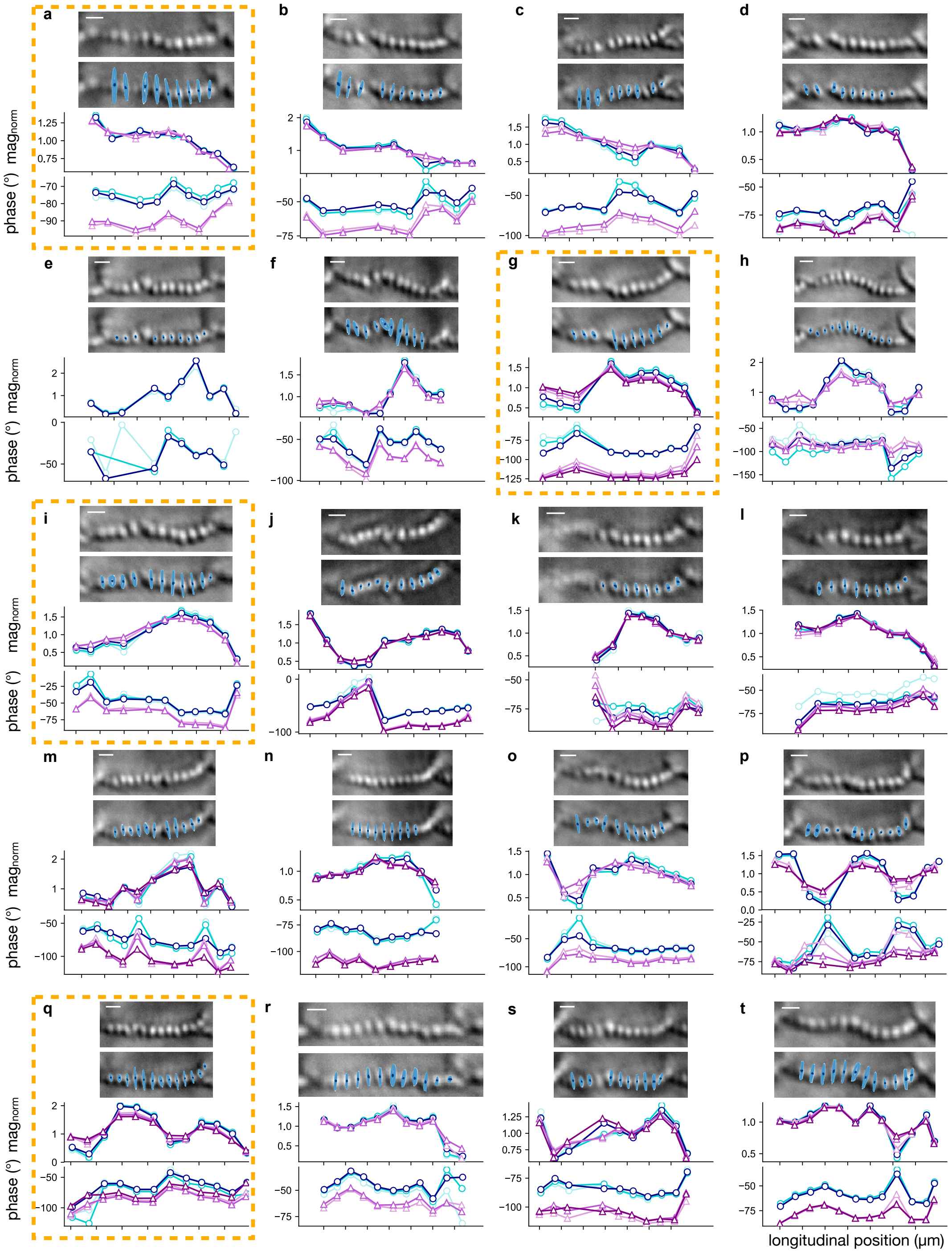

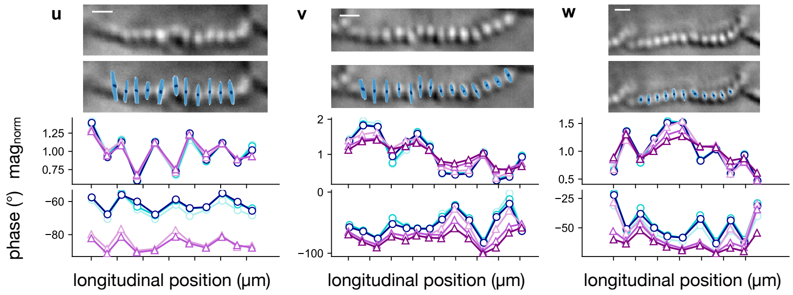

Figure S6. Motion patterns for all bundles. a–w Each panel shows an image of a bundle with a 1-μm scale bar (upper image), a copy of the same image with superimposed motion trajectories (10x) for 2-V 2-kHz stapes stimulation (lower image), and a plot of the motion magnitudes (upper plot) and phases (lower plot) relative to the apical surface, with the magnitude normalized by the average magnitude of the stereocilia in the same bundle. The 2-kHz and 3-kHz results are plotted in various shades of blue and purple, respectively, with the shade darkening as the input amplitude increases. Panels a,g,i show the bundles in Figure 3, and q shows the bundle in Figure 2.

Bundle motion relative to apical-surface motion in the longitudinal direction. As in the main text, bundle motion is defined as the average motion of the first-row stereocilia at the highest depth measured (closest to the tips). Note that the bundle magnitude is the magnitude of the mean motion of all stereocilia, which is different from the mean magnitudes of all stereocilia as presented in Figure S4. The former takes the mean first, while the latter takes the magnitude first, and thus in the latter case the mean of the magnitudes is always greater than zero. The raw bundle motion in the longitudinal (x) direction closely follows the AS motion for both dish and stapes stimulation (Fig. S7). This is evident in the similar magnitudes and phases between the raw bundle motion and AS motion (Fig. S7a–d), which causes the relative bundle motion in the longitudinal direction to be close to zero (Fig. S7a,b, right-hand panels). As such, the phase and pivot angle for the relative bundle motion in the longitudinal direction are not reported, as the calculations contain large errors.

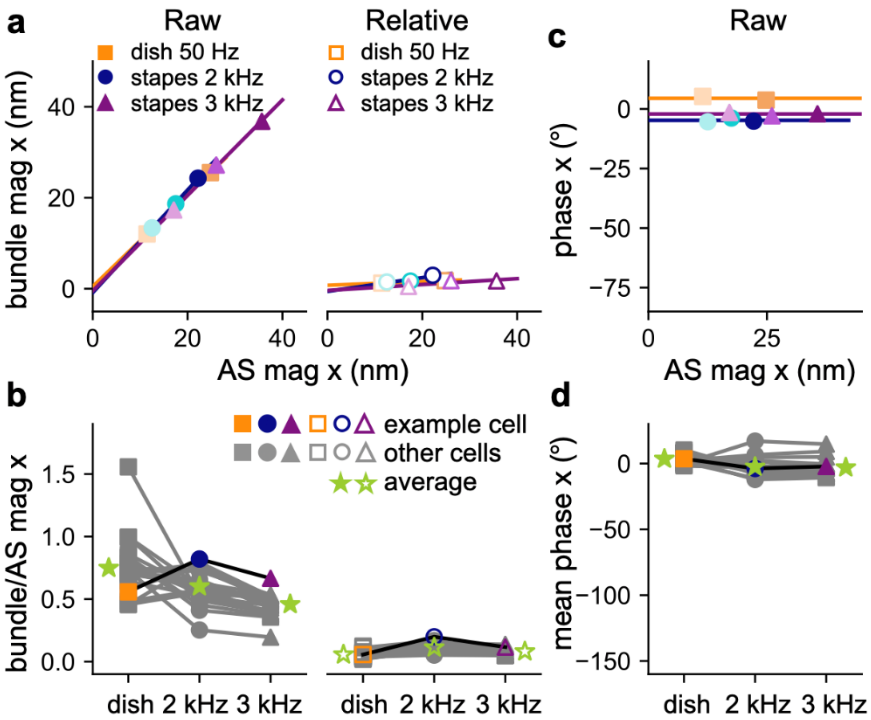

Figure S7. Bundle motion relative to apical-surface motion in the longitudinal (x) direction. Bundle motion is defined as the average motion of the first-row stereocilia. **a** For an example cell, the longitudinal bundle magnitude vs. AS magnitude is shown, with the raw motion on the left and relative motion on the right. **b** The ratios of the bundle and AS magnitudes for all cells are compared among dish stimulation and stapes stimulation at 2 and 3 kHz, with the raw motion on the left and relative motion on the right. The results for the example cell are indicated in (b) and (d) with a solid black line and colored symbols, and the average values for the ensemble are indicated by green stars. **c** For the example cell, the raw longitudinal bundle phase is plotted against the longitudinal AS magnitude. The bundle phase is relative to the AS phase. **d** The bundle phase for each cell, averaged across stimulation levels, is compared among the three stimulation types.

Supplementary Methods

Maintaining tissue integrity.

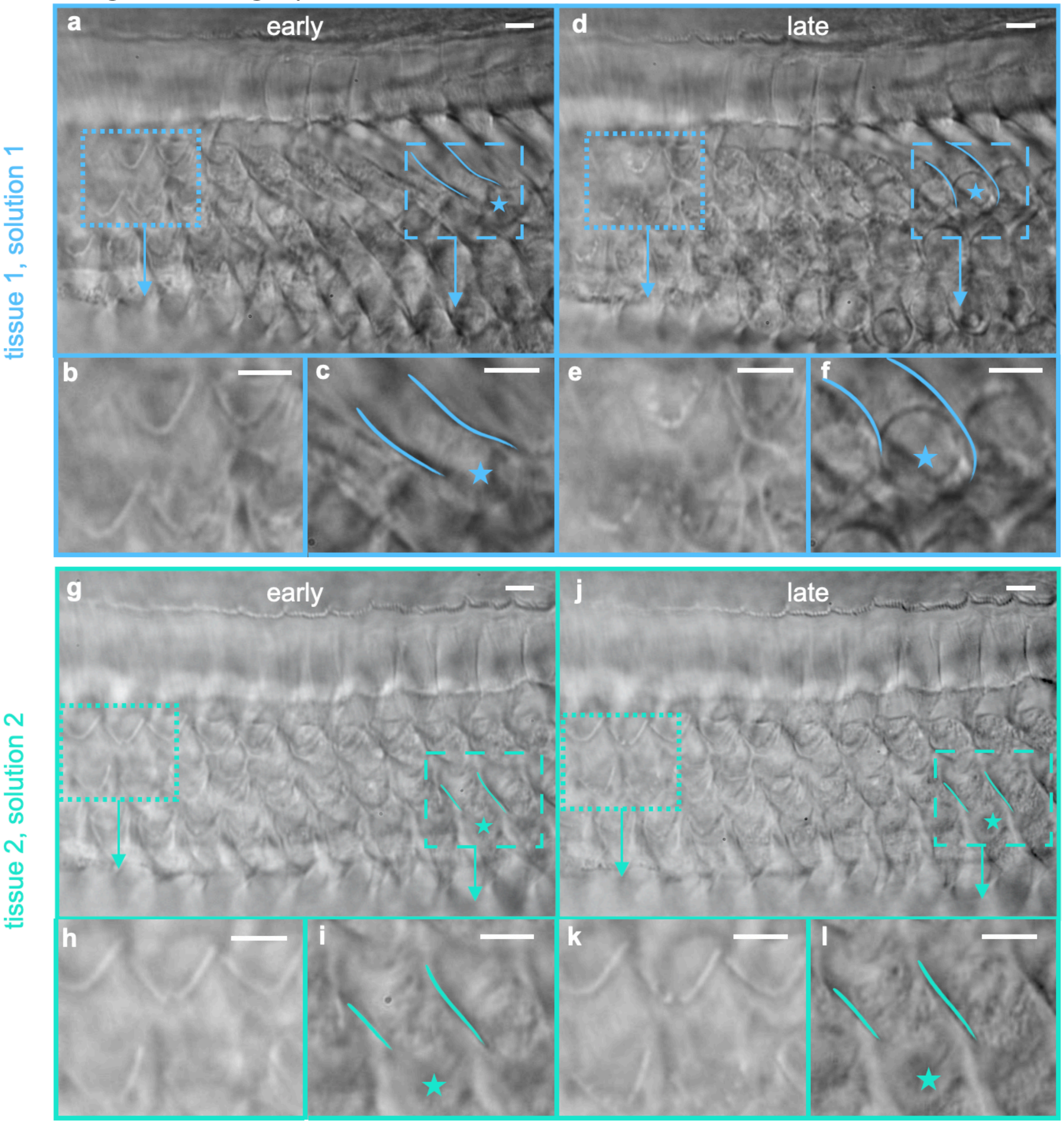

Figure S8. The effects of artificial-perilymph formulation on tissue integrity. a–f In this first example, the tissue was prepared in artificial perilymph with no amiloride, and with 2-mM $CaCl_2$. The leftmost images (a–c) depict the tissue right after dissection (a; with a 5-µm scale bar), with (b) and (c) below representing enlarged views (with a 5-µm scale bar) of the two rectangular regions in (a). This arrangement of displaying a larger image on top accompanied by two enlarged subregions underneath is also used in (d–f), (g–i), and (j–l). The images of (d–f), of the same tissue region 10 minutes after dissection, exhibit disruption of the stereocilia bundles of the outer hair cells (OHCs; e vs. b), as well as swelling of the bodies of the OHCs (f vs. c). **g–l** In this second example, the tissue was prepared in artificial perilymph that contained 1-mM amiloride and 10-mM $CaCl_2$. Compared to the images immediately after dissection (j–e), those taken 30 minutes later after completing the experiments (j–l) show that tissue integrity has been maintained with this artificial-perilymph formulation. There was minimal disruption to the OHC bundles (k vs. h), and there was no apparent swelling of the bodies of the OHCs (l vs. i).

Tissue integrity of the tectorial membrane.

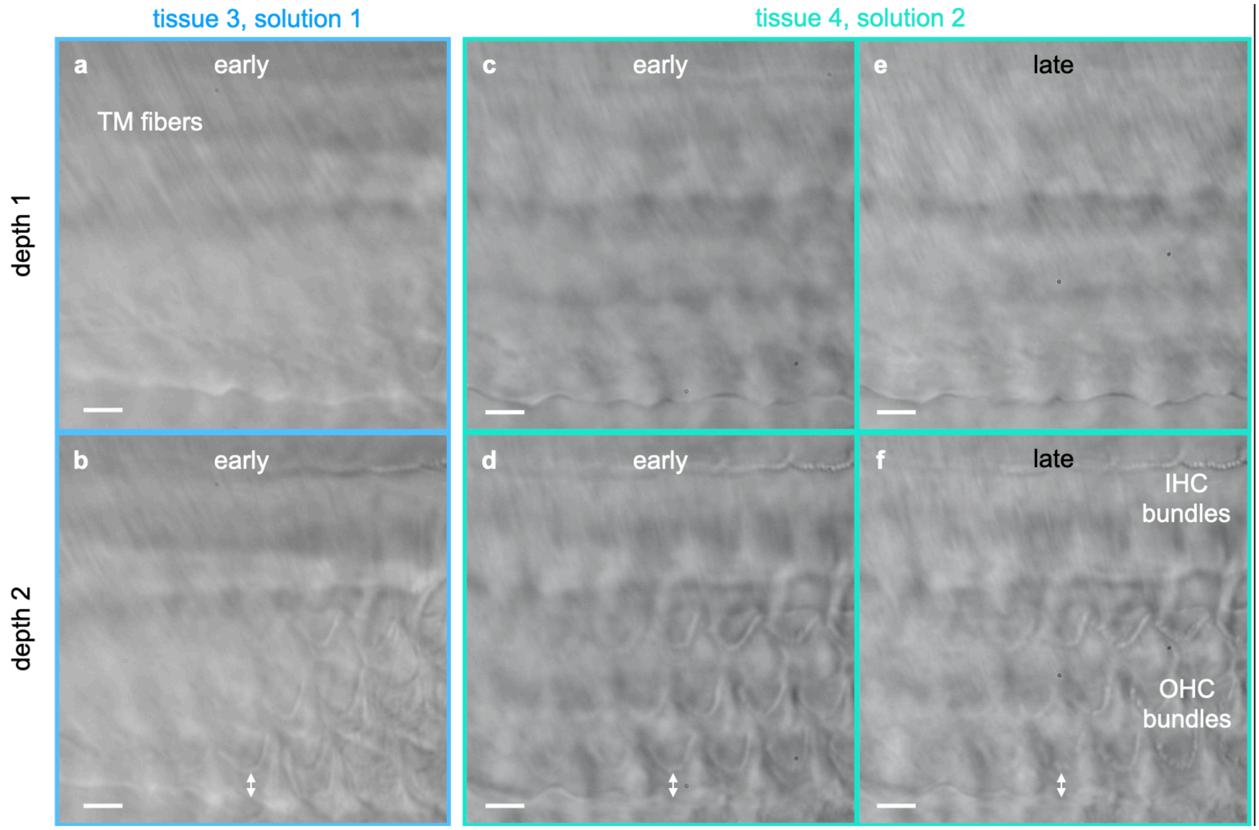

Figure S9. Comparisons of tectorial-membrane appearance at two depths in solutions with 2- or 10-mM Ca^{2+}. a–f Images of the tectorial membrane (TM) near its upper (a,c,e) and lower (b,d,f) surfaces are shown for two tissue specimens right after dissection, (a,b) and (c,d), and, for the second specimen, after completing the experiments around 30 minutes after dissection (e,f). The first specimen (a,b) was prepared in artificial perilymph with standard 2-mM Ca^{2+} without 1-mM amiloride. The second specimen was prepared in artificial perilymph with 10-mM Ca^{2+} and with 1-mM amiloride added, which is the same solution used for the experiments. The distances between the OHC bundles and the edge of the TM are indicated by white arrows, and are the same for tissues prepared in standard artificial perilymph solution 1 and for tissues prepared in solution 2 before and after the experiments. All scale bars represent 5 μm.

Estimating the equivalent ear-canal dB SPL observed at the OoC. The equivalent ear-canal pressure presented at the OoC was estimated to be 80–100 dB SPL. In order to estimate this referred pressure, we used two indirect approaches. One is to compare with experimental data, and the other is a modeling approach to estimate the pressure drop due to the holes in the cochlear wall.

For the first approach, we compared our measurements of the IHC apical-surface motion to the measurements of reticular lamina (RL) motion in the OHC region of intact postmortem cochleae from Lee *et al.*[1]. We calculated the equivalent dB SPL at the ear canal based on their measurement and on the fact that postmortem cochlear responses are known to be linear. The results are listed in Table 1, and the equivalent dB SPL at the ear canal estimated using this method is thus 80–105 dB SPL. Note that the data presented in Table 1 does not correct for the ~20° difference in the definition of the radial direction as compared to the present study, which would further reduce the estimated dB SPL by about 7 dB.

		Displacement Magnitude		Measured or equivalent SPL	
		Lowest	Highest	Lowest	Highest
Lee *et al.* (2016): RL motion, Fig. 3H&K	*y*, 2–3 kHz		10 nm		80 dB SPL
Current data: IHC apical-surface motion	*y*, 2 kHz	10 nm	88 nm	80 dB SPL	99 dB SPL
	y, 3 kHz	50 nm	188 nm	94 dB SPL	105 dB SPL

Table S1. Estimation of an equivalent ear-canal dB Sound Pressure Level based on experimental reticular-lamina motion in the radial direction as measured by Lee *et al.* (2016).

For the second approach, we built a simple 3D cochlear box model in Mathematica. The model is illustrated in Figure S10a, and the pressure distributions in the scala vestibuli (SV) within the cochlea, with and without holes, are plotted in Figure S10b.

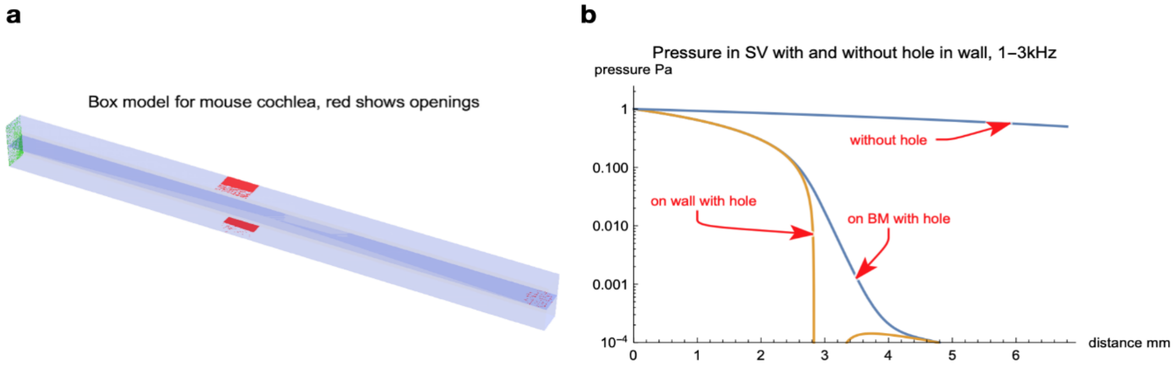

Figure S10. Pressure distributions in the mouse cochlea with and without holes, solved using a box model. a Illustration of the box model. **b** Pressure distribution in the cochlea with and without holes. Blue - pressure on the basilar membrane (BM); yellow - pressure on the cochlear wall.

With the holes, the pressure dropped quickly to zero when moving past the holes in the apical direction. At the location of the holes, the pressure dropped about 40–60 dB SPL across the area of the hole as compared to the intact cochlea. Since the equivalent ear-canal pressure reaching the stapes is estimated to be 136–156 dB SPL, the equivalent ear-canal pressure reaching the OoC with the two holes is estimated to be ~86–106 dB SPL, or -50 dB SPL relative to the equivalent pressure at the stapes.

In conclusion, based on both approaches, the equivalent ear-canal dB SPL for a passive cochlea at the location of the experiment was estimated to be approximately 80–100 dB SPL. In addition to the equivalent dB SPL for postmortem cochleae, it is important to note that in living cochleae with active amplification processes, the RL can vibrate at magnitudes over 100 nm in the radial direction for ear-canal pressures of 80 dB SPL (Lee *et al.*, 2016), and thus the motion magnitudes of the OoC observed in the current experiments are well within the normal physiological range.

Comparing Gaussian 1D fit and Fiji ImageJ plugin TrackMate. To demonstrate that the type of image-processing algorithm does not affect the sensitivity and precision of the measurements, we compare the results of TrackMate in one direction to that of the Gaussian 1D fit in the same direction (radial) on individual stereocilia from six randomly chosen samples collected from the present work. Since the sensitivity of the motion detection for stereocilia within a bundle is examined using the dish-stimulation control experiment as described earlier, here only one stereocilium was chosen from each sample for comparison. The results for an example stereocilium in the time and frequency domain are shown in Figure S10a and b, respectively, and the summary plots for comparing the magnitudes and phases are shown in panels c and d respectively. Panel c plots the magnitude of motion as measured by the Gaussian 1D fit against the magnitude obtained using TrackMate and shows a strong correlation between the two methods. Fitting the data to a line (black line) provides a slope of 0.98 ± 0.01 and an intercept of 0.06 ± 0.81 with an R^2 of 0.994. The percentage differences in motion magnitude between the two methods have a mean of 1% with a standard deviation of 5%. Panel d plots a histogram of the phase differences between the two methods and a fitted normal distribution with a peak and variance of 0.002 ± 0.02 radians, hereto suggesting no systematic difference between the two methods.

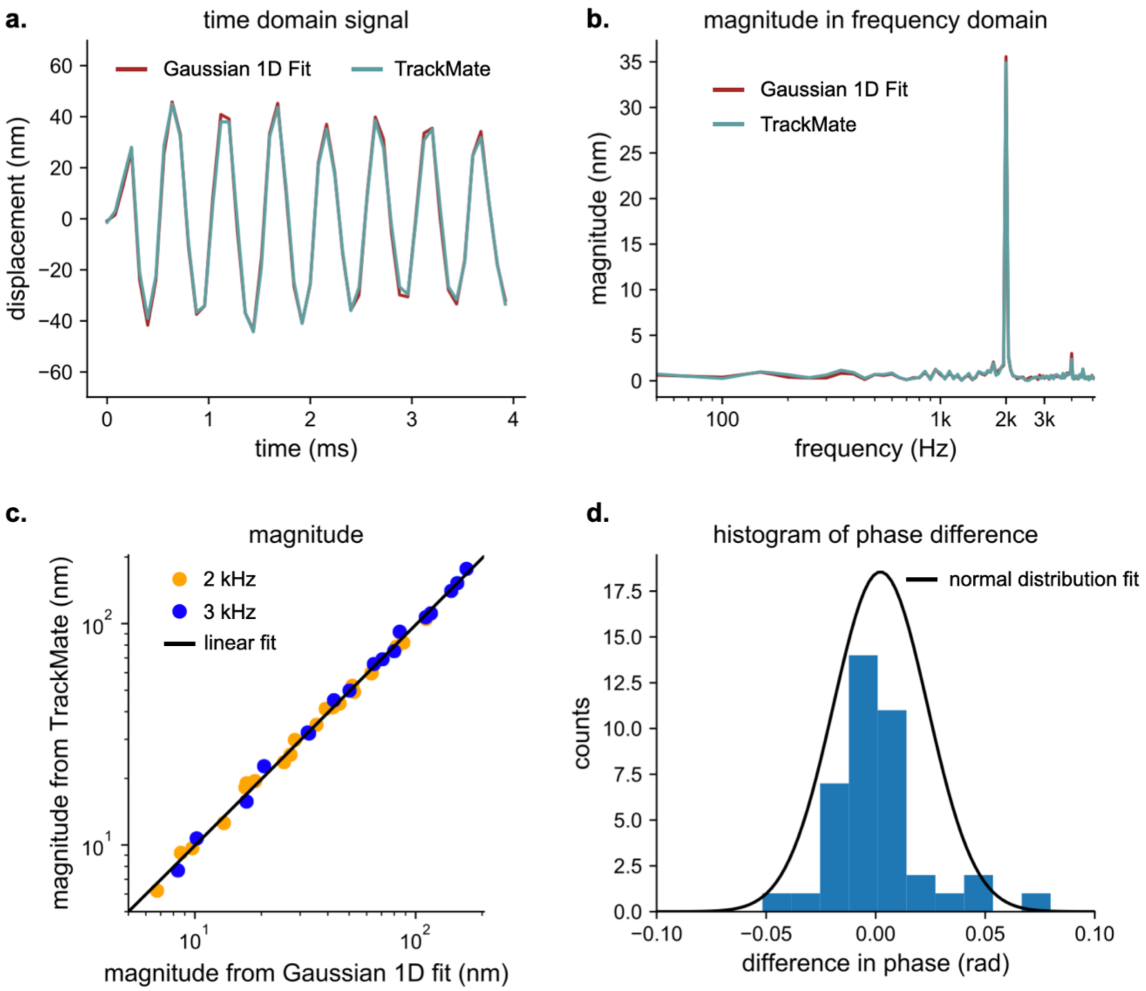

Figure S11. Comparison between results from Gaussian 1D fit and TrackMate. a Example results of a time-domain signal from one stereocilium and one stimulation run at 2 kHz. **b** Example results of the frequency-domain signal for the same stereocilium and stimulation run in a. **c** Magnitudes measured by TrackMate plotted against those measured by Gaussian 1D Fit. A linear fit (black line) shows a slope of 0.98 ± 0.01 and an intercept of 0.06 ± 0.81 with an R^2 of 0.994. **d** Histogram of the phase differences between the results measured by the two methods, with a superimposed Gaussian normal distribution.

Calculating the circular standard deviation (cSD) of the phase. To obtain the phase difference between two time-domain signals, for example between cell-body and stereocilium motion, the two signals were each broken into ten equal segments, and the Fast Fourier Transform (FFT) of each of the last eight segments was then used to determine the phase relationship between each pair of segments. When obtaining the phase difference for each pair of segments, the phase components of the complex amplitudes at all of the frequency bins of one segment were respectively normalized by those of the other segment, which for respective phases θ_1 and θ_2 amounts to $\exp[j\theta_1]/\exp[j\theta_2] = \exp[j(\theta_1-\theta_2)]$.

For calculating the mean and SD (i.e., cSD) of the eight phase differences, a standard method in circular statistics was used, as is outlined below. Note that the same method was also used for calculating the mean and cSD of the phase between stereocilia and between bundles. Using the

eight segments as an example, each phase difference was represented by a unit vector in the complex domain, and the eight unit vectors were averaged in the complex domain to obtain an averaged vector. The phase of the averaged vector was then taken as the mean of the phases. The magnitude of the averaged vector is related to the variance among the eight phase differences, with a magnitude of 1 indicating that all eight vectors are perfectly aligned, and a magnitude close to 0 indicating that the vectors are randomly distributed with respect to one another. The cSD of the phase is therefore defined as $\log(1/R^2)$, where R is the magnitude of the averaged vector. Thus, the cSD of the phase can have values ranging from 0 to infinity, and is unitless.

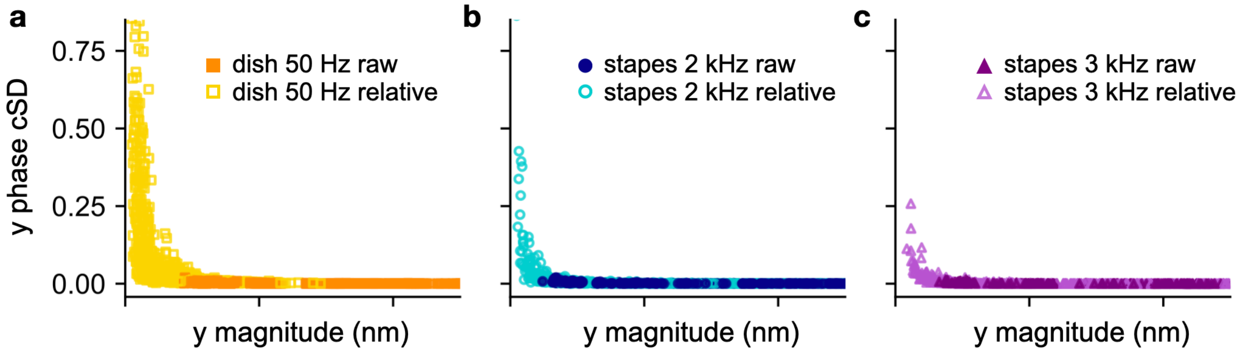

Figure S12. Circular standard deviations of the radial phase across eight-segment groups. a–c The cSDs of the radial stereocilium phase across segments are plotted against the radial motion magnitude, for dish stimulation (a), stapes stimulation at 2 kHz (b), and stapes stimulation at 3 kHz (c). This cSD calculation is a confidence indicator for the phase measurements, in which larger values indicate less confidence in the measurement. The relationship between the radial phase cSD across segments and the radial magnitude is essentially the same for all three stimulation modes, as well as between raw and relative motions. The cSD grows rapidly when the motion magnitude diminishes toward zero.

Bone motion.

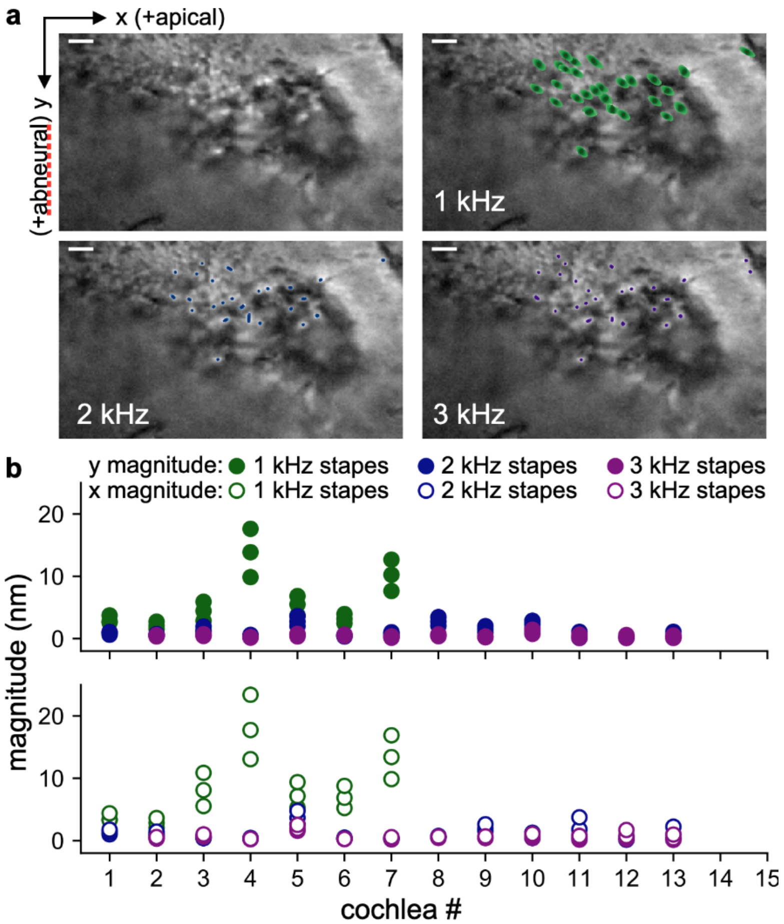

Figure S13. Bone motion. a An image of the cochlear bone from an example specimen is shown in the upper-left panel, with the scale bar representing 1 μm. The same image is reproduced in the other three panels with overlaid trajectories (scaled 15x) of the spots picked by the motion-detection algorithm for 1.6-V piezo-electric stimulations at the stapes probe at 1 kHz (upper right), 2 kHz (lower left), and 3 kHz (lower right). Similar to the way cell-body motion is calculated, the bone motion is approximated as the average motion of the detected spots on the bone. **b** The measured bone-motion magnitudes are shown across cochleae for each stimulation frequency, with the upper panel containing the radial (y) results and the lower panel containing the longitudinal (x) results. Each symbol represents one stimulation, color-coded according to the stimulation frequency. For all of the specimens in which 1-kHz measurements were taken (#1–7), the cochlear bone moved significantly more at 1 kHz than at 2 or 3 kHz, which is why that frequency was excluded from the rest of the study.

Determination of the recording depth.

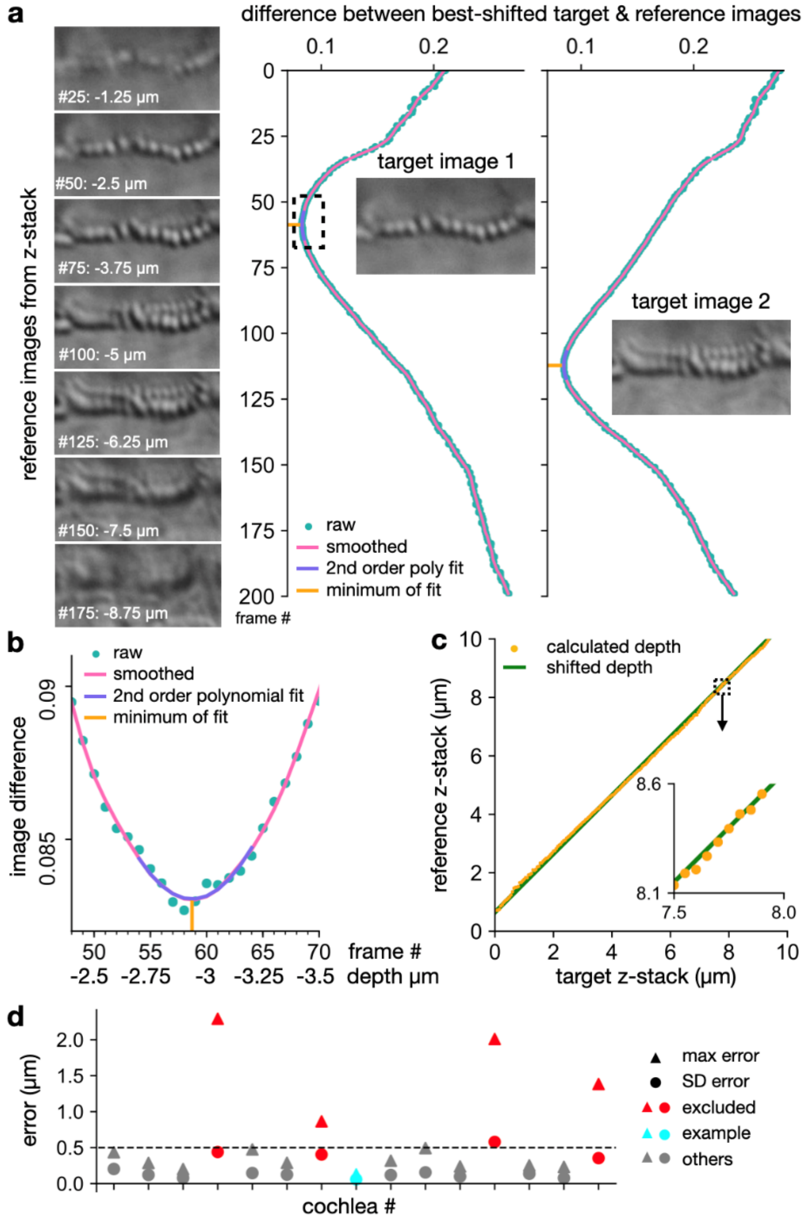

Figure S14. Determination of the recording depth. a A subset of images from a reference z-stack, at known depths, is shown on the left. For example target images 1 (middle) and 2 (right), the differences between the target image and the z-stack of reference images are plotted as functions of the z-stack frame # (vertical axis), with the raw difference calculation for each frame plotted as a blue dot, the smoothed data as a pink line, and a second-order polynomial fit around the minimum of the smoothed data (± 5 frames) shown in purple. The location of the minimum difference is indicated by an orange line. **b** Here, a rotated enlargement of the dashed box in (a) is plotted, with the image difference now shown on the vertical axis and the frame # and corresponding depth now shown on the horizontal axis. **c** The calculated depths in terms of the reference z-stack (vertical axis) are plotted against the depth within the target z-stack (horizontal axis) using orange dots. A 45° green line, shifted to best-fit the set of orange dots, indicates how much the target z-stack has shifted vertically with respect to the reference z-stack. An enlargement of the dotted box is shown in the inset. **d** The maximum error (triangles) and SD of the error (circles) from the depth-determination function are plotted for each cochlea.

References:

1. Lee, H. Y. *et al.* Two-Dimensional Cochlear Micromechanics Measured In Vivo Demonstrate Radial Tuning within the Mouse Organ of Corti. *J. Neurosci.* **36**, 8160–8173 (2016).
