# Supplementary material for: In situ motions of individual inner-hair-cell stereocilia from stapes stimulation in adult mice
Source: Commun Biol. 2021 Aug 11;4:958. doi: 10.1038/s42003-021-02459-6 (PMC8357788; doi:10.1038/s42003-021-02459-6)
Supplement: Supplementary file 2 — Reporting Summary [file 42003_2021_2459_MOESM2_ESM.pdf]

nature research

Corresponding author(s): Anthony J. Ricci

Last updated by author(s): Jun 28, 2021

Reporting Summary

Nature Research wishes to improve the reproducibility of the work that we publish. This form provides structure for consistency and transparency in reporting. For further information on Nature Research policies, see our Editorial Policies and the Editorial Policy Checklist.

Statistics

For all statistical analyses, confirm that the following items are present in the figure legend, table legend, main text, or Methods section.

n/a	Confirmed	
☐	☒	The exact sample size (n) for each experimental group/condition, given as a discrete number and unit of measurement
☐	☒	A statement on whether measurements were taken from distinct samples or whether the same sample was measured repeatedly
☐	☒	The statistical test(s) used AND whether they are one- or two-sided<br>*Only common tests should be described solely by name; describe more complex techniques in the Methods section.*
☒	☐	A description of all covariates tested
☒	☐	A description of any assumptions or corrections, such as tests of normality and adjustment for multiple comparisons
☐	☒	A full description of the statistical parameters including central tendency (e.g. means) or other basic estimates (e.g. regression coefficient) AND variation (e.g. standard deviation) or associated estimates of uncertainty (e.g. confidence intervals)
☒	☐	For null hypothesis testing, the test statistic (e.g. F, t, r) with confidence intervals, effect sizes, degrees of freedom and P value noted<br>*Give P values as exact values whenever suitable.*
☒	☐	For Bayesian analysis, information on the choice of priors and Markov chain Monte Carlo settings
☒	☐	For hierarchical and complex designs, identification of the appropriate level for tests and full reporting of outcomes
☐	☒	Estimates of effect sizes (e.g. Cohen's d, Pearson's r), indicating how they were calculated

Our web collection on statistics for biologists contains articles on many of the points above.

Software and code

Policy information about availability of computer code

Data collection	JClamp v.29.2, Phantom Camera Control (PCC)
Data analysis	Fiji ImageJ Trackmate, Python 2.7 with package SciPy, NumPy, pandas, scikit-image, Matplotlib. Mathematica 12.

For manuscripts utilizing custom algorithms or software that are central to the research but not yet described in published literature, software must be made available to editors and reviewers. We strongly encourage code deposition in a community repository (e.g. GitHub). See the Nature Research guidelines for submitting code & software for further information.

Data

Policy information about availability of data

All manuscripts must include a data availability statement. This statement should provide the following information, where applicable:

- Accession codes, unique identifiers, or web links for publicly available datasets
- A list of figures that have associated raw data
- A description of any restrictions on data availability

The data sets generated during and/or analysed during the current study are available from the corresponding author on reasonable request.

Field-specific reporting

Life sciences study design

All studies must disclose on these points even when the disclosure is negative.

Sample size	We are trying to study whether the stereocilia within one bundle move semi-indepedently. We observed semi-independent motion pattern in all 23 cells. Comparing to control experiments, the p-value is smaller than 0.001.
Data exclusions	We excluded samples whose results with the same stimulation protocol at the beginning of the experiment cannot be reproduced at the end of the experiment. For those samples, the motions at the end of the experiment were close to zero, indicating tissue degradation or a shift in position of the stapes happened during the experiment.
Replication	The motion pattern for one sample were high consistent across levels and the two frequencies tested. The result with protocol performed at the beginning of the experiment was reproduced at the end of the experiment with the same protocol. Same result is not shown in control experiments.
Randomization	This is not relevant to our study, as we did not have different groups.
Blinding	This is not relevant to our study, as we did not have different groups.

Reporting for specific materials, systems and methods

We require information from authors about some types of materials, experimental systems and methods used in many studies. Here, indicate whether each material, system or method listed is relevant to your study. If you are not sure if a list item applies to your research, read the appropriate section before selecting a response.

Materials & experimental systems

n/a	Involved in the study	
☒	☐	Antibodies
☒	☐	Eukaryotic cell lines
☒	☐	Palaeontology and archaeology
☐	☒	Animals and other organisms
☒	☐	Human research participants
☒	☐	Clinical data
☒	☐	Dual use research of concern

Methods

n/a	Involved in the study	
☒	☐	ChIP-seq
☒	☐	Flow cytometry
☒	☐	MRI-based neuroimaging

Animals and other organisms

Policy information about studies involving animals; ARRIVE guidelines recommended for reporting animal research

Laboratory animals	Mice FVB P19-21 of either sex
Wild animals	Current study did not involve wild animals
Field-collected samples	Current study did not involve field-collected samples
Ethics oversight	Methods used in current study are approved by the Stanford University Administrative Panel on Laboratory Animal Care

Note that full information on the approval of the study protocol must also be provided in the manuscript.
